# Serum concentrations of proinflammatory biomarker interleukin-6 (IL-6) as a predictor of postoperative complications after elective colorectal surgery

**DOI:** 10.1186/s12957-023-03270-9

**Published:** 2023-12-14

**Authors:** Vladimír Procházka, Lukáš Lacina, Karel Smetana, Martin Svoboda, Kateřina Skřivanová, Miroslava Beňovská, Jiří Jarkovský, Leoš Křen, Zdeněk Kala

**Affiliations:** 1 Department of Surgery, Faculty of Medicine, University Hospital Brno Bohunice, Masaryk University, Brno, Czech Republic; 2 Institute of Anatomy, First Faculty of Medicine, Charles University, Prague, Czech Republic; 3 Department of Dermatovernereology, First Faculty of Medicine, Charles University, Prague, Czech Republic; 4 Department of Clinical Psychology, University Hospital Brno-Bohunice, Brno, Czech Republic; 5 Division of Clinical Biochemistry, Department of Laboratory Medicine, University Hospital Brno, Brno, Czech Republic; 6 Department of Laboratory Methods, Faculty of Medicine, Masaryk University, Brno, Czech Republic; 7 Faculty of Medicine, Institute of Biostatistics and Analyses, Masaryk University, Brno, Czech Republic; 8 Department of Pathology, Faculty of Medicine, University Hospital Brno, Masaryk University, Brno, Czech Republic

**Keywords:** Interleukin-6, Postoperative complications, Colorectal surgery, Infection

## Abstract

**Background:**

The aim of this prospective study was to evaluate the role of serum IL-6 as a potential predictive biomarker of postoperative complications (POC) in elective colorectal surgery.

**Method:**

A total of 115 patients underwent colorectal surgery for malignancy. IL-6 was measured on the first and third postoperative days (POD1, POD3), and C-reactive protein (CRP) was measured on the POD3. POC was analysed in subgroups according to Clavien‒Dindo (CD), antibiotic (ATB) treatment, intensive care unit (ICU) and hospital length of stay. The predictive power of variables for evaluated endpoints was analysed using receiver-operating characteristic (ROC) analysis and described by area under the curve (AUC). ROC analysis was adopted for the identification of optimal cut-offs. Histological analysis was performed to verify IL-6 production by the tumour.

**Results:**

Out of 115 patients who were analysed, 42% had POC. Patients with POC had significantly higher serum levels of IL-6 on POD1 (*p* < 0.001) and POD3 (*p* < 0.001). IL-6 early on POD1 as a predictor of antibiotic treatment, ICU stay and hospital stay (*AUC* 0.818; 0.811; 0.771) did not significantly differ from the AUC of CRP late on POD3 (0.879; 0.838, 0.752). A cut-off IL-6 value of 113 pg/ml on POD1 and 180.5 pg/ml on POD3 in severe complications (CD > 3a) resulted in 75% and 72% sensitivity, 78.6% and 99% specificity, negative predictive value 96.4% and 97% and positive predictive value 29% and 88.9%.

**Conclusion:**

The serum level of interleukin-6 can predict severe (CD > 3a) POC early on POD1. On POD3, IL-6 is superior to CRP in terms of high positive predictive power of severe POC. Interestingly, the advantage of IL-6 on POD1 is early prediction of the need for antibiotic treatment, ICU stay and hospital stay, which is comparable to the CRP serum level late on the third POD.

**Supplementary Information:**

The online version contains supplementary material available at 10.1186/s12957-023-03270-9.

## Introduction

Elective colorectal surgery has relatively high postoperative mortality and morbidity due to its complexity. According to the literature, the mortality rate ranges from approximately 5 to 6%, and the morbidity rate ranges from approximately 20 to 40% [1]. Despite all improvements in surgical techniques, perioperative management and implementation of ERAS (enhanced recovery after surgery), avoidance of all postoperative complications (POC) is challenging, especially in old and fragile patients. Early detection and management of anastomotic leakage (AL) are essential to prevent severe complications and reduce mortality. These include stoma creation, prolonged length of hospital stay with negative economic aspects and peritonitis, which may lead to death. However, detection is challenging due to nonspecific signs early after operation. On the other hand, identification of patients with a low risk of POC allows early discharge with a low readmission rate. Interest in biomarkers for early prediction of AL is growing over time, and in the literature, many studies focus on different predictive systemic and peritoneal drain biomarkers. Instead of C-reactive protein (CRP), which predicts POC quite late, several studies focused on interleukin-6 (IL-6) serum levels from different points of view in colorectal surgery, but its role has not been clarified. In terms of postoperative complications, larger studies suggest that IL-6 might be helpful in predicting complications such as Clavien–Dindo (CD) > 3a, intra-abdominal infection or AL; however, conclusions differ, and some of them are rather heterogeneous. Therefore, the purpose of this study was to prospectively evaluate IL-6 in consecutive colorectal patients at our department as an appropriate biomarker to predict or detect complications early, which is an important task with multiple benefits in surgery and oncology.

There is strong evidence that many cell types produce IL-6. This cytokine is essential in the initial phase of the immune response; however, its biological impact is quite complex [2]. In the cell, IL-6 is recognized by a specific transmembrane receptor, IL-6R, which interacts with glycoprotein 130, acting as a signal transducer. IL-6 can also bind to a soluble form of the receptor (sIL-6R). This can trigger IL-6 signalling in cells not expressing IL-6R. This mechanism is usually called alternative or trans-signalling [3].

Many studies have shown the very important role of IL-6 signalling pathway activity in the biology of malignant tumours [4]. It is one of the principal mediators of the dialogue between malignant and other cells across the cancer ecosystem [5]. IL-6 serum levels are elevated in cancer patients, particularly in the advanced stages of malignancies, including colorectal cancer [6, 7]. IL-6 also participates in tumour spreading and metastasis formation in colorectal cancer [8, 9]. The elevated proinflammatory cytokines, namely, IL-1β and IL-6, can be considered markers of POC in colorectal cancer [10].

Surgical intervention inevitably elicits a proinflammatory response. However, it is also known that surgery and anaesthesia can result in a variety of metabolic and endocrine responses, which result in a generalized state of immunosuppression in the immediate postoperative period [11]. In complex surgery, it is essential to keep these two responses balanced [12]. To avoid the dramatic consequences of an overwhelming systemic inflammatory response syndrome, such as organ failure, an anti-inflammatory response (called “compensatory anti-inflammatory response syndrome”: CARS) is rapidly triggered by the host, including cortisol secretion by adrenal glands after afferent impulses from the site of injury [13, 14]. Mokart recently showed that IL-6 is a good independent early marker of postoperative sepsis, severe sepsis or septic shock after major oncological surgery [15]. The classic proinflammatory response is also activated in infectious complications, and increasing levels of inflammatory cytokines have also been reported in these complications [16].

We aimed to assess the predictive values and role of IL-6 and CRP in colorectal surgery. IL-6 serum levels might help predicting POC early and guide surgeons to provide more intensive care, prolong antibiotic (ATB) treatment and safe discharge of the patients.

## Materials and methods

### Patients

The prospective study included 122 consecutive patients operated on at University Hospital Brno Bohunice in the Czech Republic between May 2021 and September 2022. All patients were diagnosed with colorectal malignancy and underwent elective radical surgical resection with primary anastomosis (Table 1). Seven patients were excluded from a total of 122 patients due to resections indicated for inflammatory bowel disease (IBD). None of the patients was tested for Lynch syndrome. Since we started testing for microsatellite instability (MSI), 25 patients were tested. None of the patients had received immunosuppressive therapy in the last month before surgery or aspirin as a chemopreventive agent*.* All data were collected prospectively. This study was approved by the Medical Ethical Committee of the Faculty Hospital Brno (no. 10–270420/EK) in accordance with the ethical principles of the Declaration of Helsinki.

**Table 1:** Patient characteristics, POC and subgroups of patients

	*Patient characteristics (N* = *115)*
**Sex ratio (M:F)**	59 (51.3%):56 (48.7%)
**Age (years)**	68 (39–89)
**BMI (kg/m**^**2**^**)**	27 (17–43)
**Physical status classification ASA**	
1/2/3/4	1 (0.9%)/33 (28.7%)/59 (51.3%)/22 (19.1%)
**Comorbidities (overall)**	53 (46.1%)
Coronary artery disease	24 (20.9%)
Diabetes mellitus	30 (26.1%)
Thromboembolic disease	27 (23.5%)
**Rectal resections**	47 (40.9%)
TaTME	16 (13.9%)
Laparoscopic/open TME	14 (12.2%)/6 (5.2%)
Laparoscopic PME	11 (9.6%)
Operating time (min)	200 (90–380)
Adenocarcinoma	38 (33.0%)
Adenoma with HG dysplasia	4 (3.5%)
pCR after neoadjuvant CRT	3 (2.6%)
No residual malignancy after LE	2 (1.7%)
Upper/middle/distal rectum	26 (55.3%)/11 (23.4%)/11 (23.4%)
Neoadjuvant therapy	29 (61.7%)
**Colonic resections**	68 (59.1%)
Laparoscopic/open left colectomy	14 (12.2%)/6 (5.2%)
Laparoscopic/open right colectomy	23 (20.0%)/23 (20.0%)
Transverse colon resection	2 (1.7%)
Operating time (min)	195 (75–380)
Adenocarcinoma	55 (47.8%)
Adenoma with HG dysplasia	10 (8.7%)
NET	1 (0.9%)
Lymphoma	2 (1.7%)
**No complications/overall complications**	66 (57.4%)/49 (42.6%)
**Mortality**	1 (0.9%)
**Clavien‒Dindo**	
1/2	1 (0.9%)/34 (29.6%)
3a/3b	2 (1.7%)/3 (2.6%)
4a/4b	6 (5.2%)/2 (1.7%)
5	1 (0.9%)
**Reoperation**	10 (8.7%)
**Anastomotic leak**	6 (5.2%)
**Paralytic ileus**	15 (13%)
**Urinary tract complications**	8 (7%)
**Wound complications**	7 (6.1%)
**Sepsis**	4 (3.5%)
**Medical complications**	16 (13.9%)
**Subgroups of patients**	
CD > 3a	12 (10.4%)
CD > 2	14 (12.2%)
ICU (days)	4 (1; 60)
ICU > 5 days	33 (28.7%)
Hospital stay (days)	8 (6; 72)
Hospital stay > 10 days	30 (26.1%)
Antibiotic treatment	24 (20.9%)
Inflammatory complication	29 (25.2%)

*M* male, *F* female, *TaTME* transanal total mesorectal excision, *TME* total mesorectal excision, *PME* partial mesorectal excision, *LC* left colectomy, *RC* right colectomy, *pCR* pathologic complete response, *CRT* chemoradiotherapy, *LE* local excision, *NET* neuroendocrine tumour, *HG* high grade, *BMI* body mass index, *ASA* American Society of Anesthesiologists. Categorical variables are described by absolute (relative) frequencies. Continuous variables are described by median (minimum–maximum)

### Oncological and surgical management

All patients with rectal tumours were assessed by a multidisciplinary team. Staging (according to the American Joint Committee on Cancer) was performed using whole-body positron emission tomography with magnetic resonance imaging (PET-MRI) [17]. Patients diagnosed with colon tumours were staged with abdominal and chest CT (computed tomography). Rigid rectoscopy and/or colonoscopy was performed to obtain a biopsy and exclude synchronous lesions. Neoadjuvant therapy, mostly chemoradiotherapy (CRT), was indicated by the oncologist according to recent guidelines [18, 19]. Adjuvant treatment after colon resections was decided by the oncologist, according to definitive histology.

Surgery management included the ERAS protocol, assessment of sphincters if a sphincter-saving procedure was possible (patients with middle and distal rectal tumours) and mechanical bowel preparation with oral antibiotics.

The procedure for rectal resection was described in detail previously [20]. Briefly, we operated mostly laparoscopically. Total mesorectal excision (TME) was performed for the middle (5–10 cm from the anal verge) and distal (0–5 cm) tumours, and partial mesorectal excision (PME) was performed for the upper tumours (10–15 cm). The transanal approach (using GelPOINT®) was used when TME could not be completed laparoscopically. The anastomosis was either hand sewn (in case of low anastomosis) or stapled. All patients were operated on between 10 and 12 weeks after neoadjuvant CRT, and all of them had created defunctioning loop ileostomy.

For right-sided colon tumours, handsewn anastomosis was usually performed, and complete mesocolic excision was indicated according to expert consensus [21]. In the case of descending colon/sigmoid tumours, left colectomy with side-to-end colorectal anastomosis was usually performed with a circular stapler. Tumours localized in the middle of the transverse colon were managed by transverse resection with side-to-side anastomosis. Indocyanine green (ICG) fluorescence angiography was used routinely to determine anastomotic perfusion.

### Detection of IL-6 in resected tissue

The methodology of immunohistochemical analysis is available in Supplementary document no. 3.

### Postoperative observations

We assessed postoperative morbidity and mortality in the first 90 days after the operation. Postoperative morbidity was evaluated using the CD classification. In CD classification, each POC is classified into one of five categories depending on its severity [22]. For a better assessment of POC, we also created subgroups of patients who had a postoperative inflammatory complication (PIC) and who needed ATB treatment. PIC included surgical site infections (SSI), parastomal abscesses, urinary tract infections, pneumonia, peritonitis and sepsis. To be more accurate, we also recorded the need for ATB treatment after surgery since not all PIC require antibiotics (COVID-19 infection and some SSI). Hospital and intensive care unit (ICU) stays were also recorded.

### Study design

A total of 115 patients were included in the analysis. Patients were divided into six subgroups according to different characteristics, i.e. different types of complications. The subgroups consisted of the following: patients with CD > 2, CD > 3a, ICU hospital stay > 5 days and hospital stay > 10 days, patients who needed ATB treatment, and patients who suffered from PIC. Subgroups of patients with CD > 2 and CD > 3 are considered patients with major complications who require surgical, endoscopic or radiological intervention without general anaesthesia and intervention under general anaesthesia. These are the most serious, clinically relevant complications, which mainly influence the postoperative course. Patients assigned to the ATB treatment subgroup needed antibiotics in the postoperative period. The inflammatory complication subgroup consisted of patients who had incisional SSI, intra-abdominal infection (collection, abscess, peritonitis), sepsis, respiratory and urinary or intestinal infection with identification of the organism(s) by culture or non-culture-based microbiologic testing methods. We believe the aspect of ATB treatment and PIC can add additional and more precise information about postoperative course, and if predicted, it might serve as a guide for early ATB treatment. Prolonged ICU and hospital stays are connected with adverse postoperative course and were defined as ICU stay > 5 days and hospital stay > 10 days. All patients were analysed for serum levels of IL-6 on postoperative day (POD) 1 and POD3 and CRP on POD3. We hypothesized that high serum levels of IL-6 could serve as a predictive factor for POC, hospital and ICU stays and the need for ATB. The results were also analysed to set up the cut-off values of predictive factors.

### Blood sample analysis

All blood samples were drawn using routine blood tests in the morning from the peripheral or central venous system. A blood sample was routinely analysed in the hospital biochemical laboratory, where Li heparin plasma was used to determine the CRP (mg/l) and IL-6 (pg/ml) values. Automatic analyses were carried out in the clinical chemistry module c702 of Cobas 8000 (F. Hoffman-La Roche Ltd.; hereinafter, Roche) via immunoturbidimetric CRP test (Ref. 07876424 190) and in the immunochemical module e801 of Cobas 8000 via electrochemiluminescence noncompetitive IL-6 immunoassay (Ref. 07027532190).

### Statistics

Standard descriptive statistics were applied in the analysis — absolute and relative frequencies for categorical variables and median supplemented by minimum–maximum range for continuous variables. The predictive power of variables for evaluated endpoints was analysed using receiver-operating characteristic (ROC) analysis and described by area under the curve (AUC), its 95% confidence interval and statistical significance. ROC analysis was adopted to identify optimal cut-offs for continuous variables at the point of the maximum sum of sensitivity (SN) and specificity (SP). Cut-off values were obtained using the Youden index. The area under the receiver operating characteristic curve (AUROC) is a widely recognized metric in medical diagnostics. It quantifies a diagnostic test’s ability to distinguish between different conditions or groups of patients. An AUROC value of 1.0 signifies a perfect test, while 0.5 suggests that the test is no better than chance, represented as a diagonal line on the graph. An AUROC exceeding 0.9 indicates a highly effective test with strong discriminatory power [23]. To compare ROC curves, the *p*-value was computed using the method by Hanley and McNeil. Analysis was computed using SPSS 28.0.1.1 (IBM Corporation 2021), and *p* = 0.05 was adopted as the level of statistical significance in all analyses. No correction for multiple testing was applied.

## Results

### Characteristics of patients

Of the 115 analysed patients, 40.9% underwent rectal resection, mostly in the third clinical stage (61.7%), and 59.1% of patients underwent colonic resection, mostly in the second pathological stage (35.3%). Most procedures were performed laparoscopically (67.8%), and the most common pathological finding was adenocarcinoma (80.9%). Neoadjuvant therapy was administered in 29 (25.2%) patients only for rectal tumours. Six patients had MSI (5 colon, 1 rectum), 19 patients were microsatellite stable (MSS) (11 colon, 8 rectum) and 90 patients were not tested. Because of a small sample, we did not perform analysis further analysis. Detailed characteristics are given in Table 1.

### Detection of IL-6 and IL-6R in cancer samples

To verify the tumour as the source of IL-6, colorectal carcinoma from the patients was subjected to histological analysis. The detailed description and histological section specimen picture are available in Supplementary document no. 3.

### Postoperative complications

A total of 49 (42%) patients had complications, and one patient with many medical comorbidities died from septic shock after a right colectomy due to progressive small bowel ischemia, which required reoperations. The most common complication according to CD classification was grade 2, which mostly included paralytic ileus, urinary tract infection and SSI. Reoperation was indicated for all six cases of AL, and in five cases, ileostomy was created. AL occurred in four cases after right colectomy and only in two cases after rectal resections, due to higher percentage (55.3%) of upper rectal tumours (see Table 1).

### IL-6 and CRP as predictors of postoperative complications

**Fig. 1:**
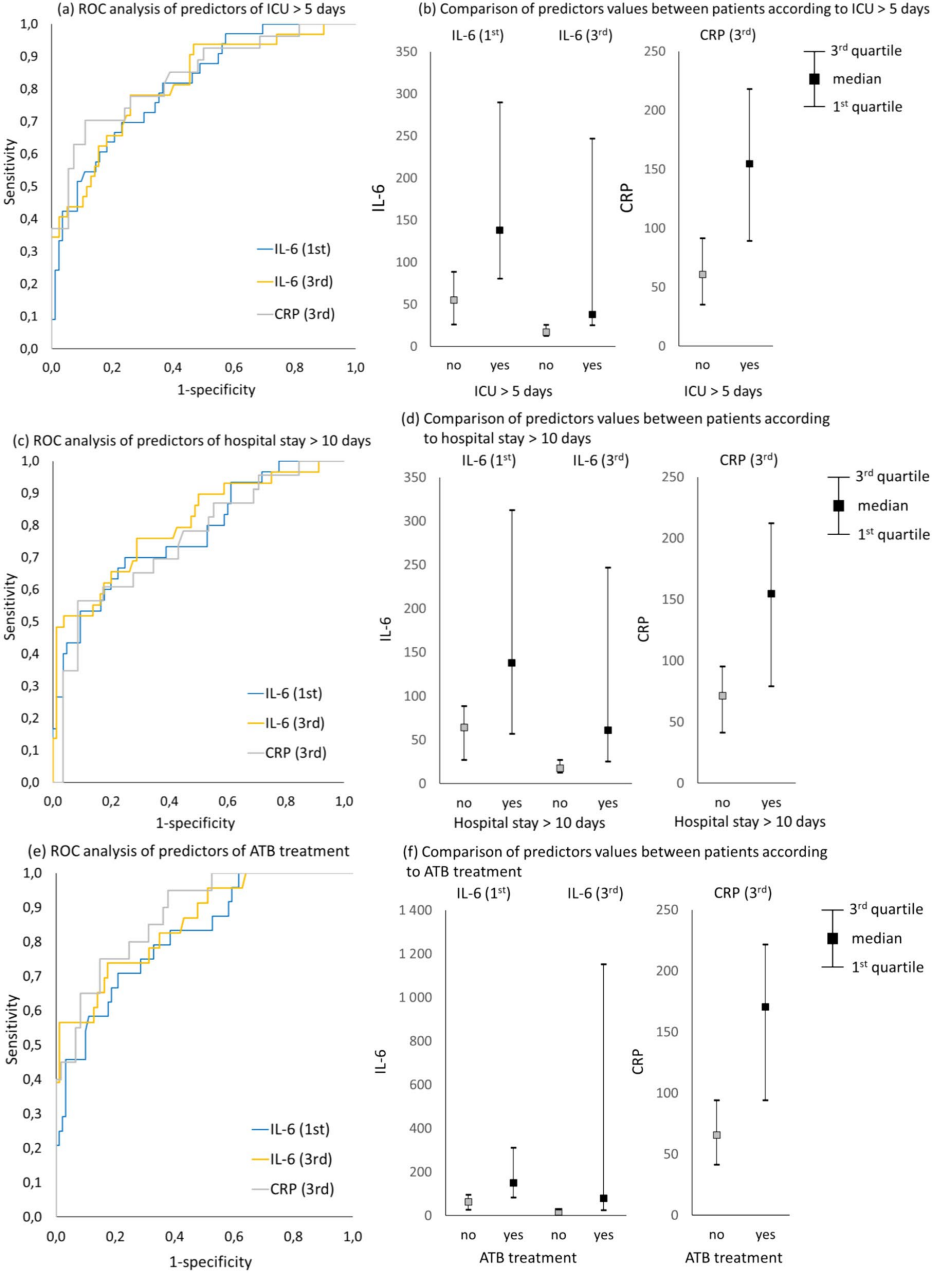
ROC curves and box plot graphs of IL-6 on POD1 and POD3 and CRP on POD3 as predictors of ICU (**a**, **b**), hospital length of stay (**c**, **d**) and ATB treatment (**e**, **f**). Axis *y* represents true-positive rate (sensitivity), and axis *x* represents false-positive rate (1—specificity). The statistical analysis and significance of the data are shown in Table 3

Biomarkers IL-6 and CRP as predictive factors are presented in Table 2. IL-6 and CRP values were significantly higher in particular subgroups of patients with POC (*p* < 0.001). The best marker in terms of greatest AUC (0.914; *CI* 0.817–1.000) was the IL-6 value on the third day after the operation, except for the prediction of ICU stay more than 5 days (*AUC* 0.814; CI 0.724–0.904) and inflammatory complications (*AUC* 0.839; 0.758–0.920), where CRP proved to be superior (*AUC* 0.838 and 0.904). Moreover, IL-6 on POD1 as a predictor of POC was statistically significant (*p* < 0.001) and, therefore, could predict complications, most accurately the need for antibiotic treatment (*AUC* 0.818; CI 0.725–0.912). No statistically significant differences were found in IL-6 levels depending on the tumour height in the rectum (Fig. 1, Supplementary documents nos. 1 and 2).

**Table 2:** Strength of predictors between patient subgroups

Predictor (POD)	Patient subgroups		AUC (95% *CI*)	*p*-value
	**Clavien‒Dindo > 3a**	**Clavien‒Dindo < 3a**		
IL-6 (1st)	*N* = 12; 189 (46; 2 746)	*N* = 103; 66 (9; 936)	0.804 (0.673; 0.934)	< 0.001
IL-6 (3rd)	*N* = 11; 1 153 (19; 50 000)	*N* = 98; 19 (3; 473)	0.914 (0.817; 1.000)	< 0.001
CRP (3rd)	*N* = 10; 192 (63; 420)	*N* = 71; 76 (14; 342)	0.869 (0.755; 0.983)	< 0.001
	**Clavien‒Dindo > 2**	**Clavien‒Dindo < 2**		
IL-6 (1st)	*N* = 14; 145 (31; 2 746)	*N* = 101; 66 (9; 936)	0.751 (0.612; 0.890)	0.002
IL-6 (3rd)	*N* = 13; 263 (18; 50 000)	*N* = 96; 19 (3; 473)	0.865 (0.755; 0.976)	< 0.001
CRP (3rd)	*N* = 10; 192 (63; 420)	*N* = 71; 76 (14; 342)	0.869 (0.755; 0.983)	< 0.001
	**ICU > 5 days**	**ICU < 5 days**		
IL-6 (1st)	*N* = 33; 138 (32; 2 746)	*N* = 82; 56 (9; 419)	0.811 (0.727; 0.895)	< 0.001
IL-6 (3rd)	*N* = 32; 43 (9; 50 000)	*N* = 77; 18 (3; 83)	0.814 (0.724; 0.904)	< 0.001
CRP (3rd)	*N* = 27; 155 (32; 420)	*N* = 54; 62 (14; 185)	0.838 (0.742; 0.934)	< 0.001
	**Hospital stay > 10 days**	**Hospital stay < 10 days**		
IL-6 (1st)	*N* = 30; 138 (26; 2 746)	*N* = 85; 65 (9; 352)	0.771 (0.669; 0.872)	< 0.001
IL-6 (3rd)	*N* = 29; 61 (8; 50 000)	*N* = 80; 18 (3; 1 387)	0.800 (0.700; 0.899)	< 0.001
CRP (3rd)	*N* = 23; 155 (32; 292)	*N* = 58; 73 (14; 420)	0.752 (0.631; 0.873)	< 0.001
	**ATB treatment**	**No ATB treatment**		
IL-6 (1st)	*N* = 24; 152 (43; 2 746)	*N* = 91; 64 (9; 352)	0.818 (0.725; 0.912)	< 0.001
IL-6 (3rd)	*N* = 23; 79 (15; 50 000)	*N* = 86; 18 (3; 114)	0.854 (0.766; 0.942)	< 0.001
CRP (3rd)	*N* = 20; 171 (63; 420)	*N* = 61; 66 (14; 201)	0.879 (0.798; 0.959)	< 0.001
	**Inflammatory complication**	**No inflammatory complication**		
IL-6 (1st)	*N* = 29; 138 (31; 2 746)	*N* = 86; 61 (9; 352)	0.798 (0.705; 0.890)	< 0.001
IL-6 (3rd)	*N* = 28; 52 (15; 50 000)	*N* = 81; 18 (3; 114)	0.839 (0.758; 0.920)	< 0.001
CRP (3rd)	*N* = 22; 178 (63; 420)	*N* = 59; 64 (14; 178)	0.904 (0.833; 0.974)	< 0.001

*POD* postoperative day, *AUC* area under the curve, *CI* confidence interval, *ICU* intensive care unit, *ATB* antibiotics. Continuous variables are described by median (minimum–maximum). *N* is the number of patients with or without specific complication. IL-6 serum levels are measured in pg/ml and CRP in mg/l. Parentheses include minimum and maximum values

We also analysed our results to set the cut-off values of each marker (Table 3). We observed that IL-6 on POD3 with a cut-off ≥ 180.5 was the strongest predictive factor in terms of POC CD > 3a, with the best combination of sensitivity (72.7%), specificity (99%), negative predictive value (NPV) (97.0%) and positive predictive value (PPV) (88.9%). Although the highest sensitivity (90%) was observed in patients with a CRP cut-off higher than 113.8 on POD3 in terms of CD > 3 and CD > 2 complications, it mostly had a negative predictive value (98.2%). The best statistically significant (*p* < 0.001) positive predictive value (88.9%) was seen in IL-6 ≥ 180.5 on POD3 in CD > 3a complications; therefore, it might be the best predictor. Interestingly, we showed that IL-6 POD1 predicts ICU and hospital length of stay and ATB treatment (*AUC* 0.733, 0.726, 0.750) comparable to CRP on POD3 (*AUC* 0.796, 0.740, 0.801); there were no statistically significant differences between ROC curves (*p* = 0.333, *p* = 0.678, and *p* = 0.813). See supplementary document no. 2.

**Table 3:** Cut-off values, NPV, PPV, sensitivity and specificity of predictors

Positive class for ROC analysis	Predictor cutoff (POD)	AUC (95% *CI*)	*p*-value	Sensitivity (%)	Specificity (%)	NPV (%)	PPV (%)
**Clavien‒Dindo > 3a**	IL-6 (1st) ≥ 113.0	0.768 (0.619; 0.918)	0.002	75.0	78.6	96.4	29.0
	IL-6 (3rd) ≥ 180.5	0.859 (0.699; 1.000)	< 0.001	72.7	99.0	97.0	88.9
	CRP (3rd) ≥ 113.8	0.844 (0.721; 0.968)	< 0.001	90.0	78.9	98.2	37.5
**Clavien‒Dindo > 2**	IL-6 (1st) ≥ 113.0	0.713 (0.558; 0.867)	0.010	64.3	78.2	94.0	29.0
	IL-6 (3rd) ≥ 34	0.808 (0.684; 0.933)	< 0.001	84.6	77.1	97.4	33.3
	CRP (3rd) ≥ 113.8	0.844 (0.721; 0.968)	< 0.001	90.0	78.9	98.2	37.5
**ICU > 5 days**	IL-6 (1st) ≥ 89.3	0.733 (0.627; 0.838)	< 0.001	69.7	76.8	86.3	54.8
	IL-6 (3rd) ≥ 25.0	0.761 (0.660; 0.862)	< 0.001	78.1	74.0	89.1	55.6
	CRP (3rd) ≥ 109.0	0.796 (0.682; 0.910)	< 0.001	70.4	88.9	85.7	76.0
**Hospital stay > 10 days**	IL-6 (1st) ≥ 89.3	0.726 (0.617; 0.836)	< 0.001	70.0	75.3	87.7	50.0
	IL-6 (3rd) ≥ 60.9	0.740 (0.619; 0.861)	< 0.001	51.7	96.3	84.6	83.3
	CRP (3rd) ≥ 146.0	0.740 (0.605; 0.874)	< 0.001	56.5	91.4	84.1	72.2
**ATB treatment**	IL-6 (1st) ≥ 104.0	0.750 (0.633; 0.866)	< 0.001	70.8	79.1	91.1	47.2
	IL-6 (3rd) ≥ 34.2	0.782 (0.668; 0.897)	< 0.001	73.9	82.6	92.2	53.1
	CRP (3rd) ≥ 113.8	0.801 (0.679; 0.924)	< 0.001	75.0	85.2	91.2	62.5
**Inflammatory complication**	IL-6 (1st) ≥ 136.5	0.729 (0.610; 0.848)	< 0.001	55.2	90.7	85.7	66.7
	IL-6 (3rd) ≥ 34.0	0.753 (0.640; 0.865)	< 0.001	67.9	82.7	88.2	57.6
	CRP (3rd) ≥ 113.8	0.827 (0.714; 0.940)	< 0.001	77.3	88.1	91.2	70.8

*POD* postoperative day, *AUC* area under the curve, *CI* confidence interval, *NPV* negative predictive value, *PPV* positive predictive value, *ICU* intensive care unit, *ATB* antibiotics. Continuous variables are described by median (minimum–maximum). Cut-off values for IL-6 serum levels are measured in pg/ml and CRP in mg/l. Parentheses include minimum and maximum values

## Discussion

The main purpose of ERAS in colorectal surgery is to reduce postoperative stress and to allow faster return of physiological functions and shorter hospital stay. To achieve these statements, postoperative complications must be detected early to prevent morbidity and mortality, and this remains a problem. In the literature, many studies focus on different predictive systemic and peritoneal drain biomarkers (inflammatory, microbiological, markers of ischemia) and frequently mention CRP, procalcitonin (PCT), neutrophil-to-lymphocyte ratio, white blood cell count and IL-6. Additionally, changes in serum albumin, nutritional parameters and sarcopenia have been studied as predictive preoperative biomarkers to detect high-risk patients before surgery [24, 25].

One of the most investigated biomarkers, which has also changed management in colorectal surgery, is CRP, displaying high sensitivity for infectious POC with a high NPV. According to a multicentric PREDICT study, a change in the CRP level exceeding 50 mg/l between any two PODs can accurately rule out AL (*NPV* 99%). Moreover, CRP monitoring in patients after TME can predict safe discharge on POD5 [26–28]. Nevertheless, a specific cut-off value to definitely rule out AL is not yet clear, and values range from 94 to 190 ml/l at different time points from POD3 to POD5. Therefore, some studies suggest monitoring the trajectory of biomarker changes, as it might be more accurate [29]. In addition, corticosteroids and statins may decrease CRP [30]. The drawback of CRP is that it predicts POC quite late (from POD3, improving later after operation) and usually has only a strong NPV. Our values of CRP on POD3 were consistent with those of known studies. In our subgroups, CRP allowed us to rule out infectious complications, the need for ATB treatment (*AUC* 0.904, 0.879) and serious POC CD > 3a, which included all cases of reoperation for AL (*AUC* 0.869). Cut-off values ranged from 109 to 146 mg/l between the observed categories of complications. It is worth to mention that Holmgren et al. described elevation of preoperative CRP in patients with AL after colonic resection but not rectal resection. They also found that patients with AL after rectal resections had significantly elevated preoperative serum levels of inflammation-related proteins CXCL6 (C–X–C motif chemokine ligand 6) and CCL11 (C–C motif chemokine ligand 11) [31].

Serum cytokine IL-6 has been investigated as a predictor of early detection of postoperative sepsis [15]. However, homogenous data on serum IL-6 and POC and early discharge after colorectal surgery seem to be limited. The promising advantage of IL-6 is its ability to detect POC early after surgery. Rettig et al. studied 137 patients after major abdominal surgery and found that POC is associated with high IL-6 serum levels on POD1. Only 67 patients (48.9%) underwent colorectal surgery [16]. We confirmed this conclusion in our study on POD1 as well as POD3 (*p* < 0.001). Serum IL-6 as a predictor of AL leak in colorectal surgery was investigated in several studies [32–35]. In these four studies, the main limitations are small samples (range 22–84 patients), different sampling times and different analytical methods. This could also be a problem in our study; therefore, we did not analyse AL alone. In detail, Reisinger et al. showed no correlation between IL-6 and POC but did not demonstrate any data on IL-6. Ellebæk et al. showed an increased median IL-6 level in patients with and without AL (a total of 26 patients, 4 had AL) on POD1 compared to the preoperative level but without any statistical analysis. Similar results were seen by Slotwińsky et al. in 22 patients with no statistical significance of IL-6 between the groups without and with postoperative infectious complications. Conversely, Alonso et al. studied the relationship between intra-abdominal infection and tumour recurrence and showed higher serum IL-6 in patients with AL or intra-abdominal abscess on POD2 and POD4 (*p* = 0.014, 0.009) and in patients with recurrence (*p* < 0.05). These significant outcomes are probably due to a better study design (30 patients with complications vs. 30 patients without). Our results support a correlation between IL-6 and not only infectious complications but also CD > 3a and CD > 2 on POD1 and POD3. Interestingly, we found that patients with the need of ATB treatment and longer ICU and hospital length of stay had significantly higher IL-6 serum levels (*p* = 0.001).

The following studies improved the statistical analysis and focused on IL-6 as a potential early predictor of complications. Boersema et al. in 2018 found that in 47 patients after colorectal surgery, the serum IL-6 ratio (preoperative/POD) cannot predict postoperative ileus but can predict infectious complications on POD1 and POD 3 with a larger AUC than CRP (0.825 and 0.801 vs. 0.732 and 0.731) [36]. We cannot properly compare these results with those in our study since a ratio was used. Interesting data presented by Zawadzki et al. focused on 32 patients with rectal tumours, and they found that IL-6 on POD3 can predict AL (*p* < 0.001, *AUC* 0.82, cut-off > 65.9 pg/ml, *SN* 100% and *SP* 76%, *PPV* 31, *NPV* 100), but preoperative IL-6 cannot (*p* = 0.286). Moreover, changes in IL-6 were not affected by the type of surgical approach (robotic or open) or the length or extent of surgery; however, only five patients had AL [37]. Consistent with this study, we also found that IL-6 on POD3 was very strong, particularly in the detection of POC CD > 3a (*p* < 0.001, *AUC* 0.85, cut-off > 180.5, *SN* 72% and *SP* 99, *PPV* 88, *NPV* 97). Surprising is the fact that on POD3, IL-6 has a high PPV (88%) of serious complications (CD > 3a) and is thus superior to CRP, which has dominantly NPV. In affected patients, this might provide a clinical implication in terms of more intensive care, control abdominal CT scan, or escalation/prolonged ATB treatment. However, these implications need to be further studied before introduction into clinical practice.

Two studies combined patients with colorectal cancer and benign disease (IBD, diverticulosis and others), and the results were inconclusive. Zielińska-Borkowska et al. are the only study that reported no predictive value of IL-6 for AL on POD1 in uni- and multivariate analyses (*p* > 0.05, *AUC* 0.61). This was a prospective study in a total of 157 patients; however, 36% of patients had a benign disease, and no information about anti-inflammatory drugs was given [38]. In the second study by Sammour et al., of a sample of 206 patients, 35% had benign disease, and IL-6 on POD1 was significant in detecting AL (*p* = 0.048, *AUC* 0.65); however, the use of anti-inflammatory drugs was not recorded [39]. A study that focused on IL-6 as a predictor of intra-abdominal septic complications (AL, abscess, fistula) in 118 patients with only Crohn’s disease found IL-6 as a significant predictor of POC on POD1, POD3 and POD5 (*p* < 0.001 for all, *AUC* 0.71, 0.86, 0.82), and 45% of patients had anti-inflammatory treatment [40]. There is an evident discrepancy between studies when detecting POC on POD1. It seems that IL-6 as a predictive factor for benign disease is affected by medication and might not be as accurate as for patients with colorectal tumours. Our study adds evidence that IL-6 on POD1 is capable of predicting POC in colorectal patients with high NPV.

Finally, some large studies also exist. In a Danish study, [40] authors analysed 401 patients divided by age (210 old and 191 young, threshold 70 years), where preoperative high levels of IL-6 but not CRP in the old were associated with major complications (CD > 3a). On POD1, a twofold increase in IL-6 predicted major complications only in patients < 70 years, and on POD3, a twofold increase in IL-6 from preoperative levels predicted major complications in both age groups (OR (odds ratio) = 1.75, 1.24–2.46, *p* = 0.002) [41]. A possible explanation for the age difference could be increased IL-6 levels in older and malnourished patients [8, 42–45]. In a multicentric prospective New Zealand study, Su’a et al. analysed 283 patients who underwent only colonic surgery (no rectum) and showed [46] a statistically significant difference between AL and no AL on POD 1 (*AUC* 0.68, *p* = 0.03, a cut-off value of 10.8 pg/mL gave an NPV of 99.1%, sensitivity 0.85, specificity 0.83) and POD 2 (*AUC* 0.69, *p* = 0.02). The explanation for low AUC and cut-off values compared to our study is probably the fact that these authors combined benign and malignant diagnoses as discussed before, and the ratio between those is unavailable. Nevertheless, the conclusions of these two studies support our results that measuring IL-6 postoperatively has a potential benefit in predicting POC.

There are some limitations of this study. We selected complications in six different subgroups as mentioned in methodology due to a small sample size, and therefore, we did not analyse anastomotic leakage alone. Also, there were some missing data (samples), which might have biased the results. Our study also lacks external validation.

## Conclusion

In this study, we found that the serum level of interleukin-6 can predict severe (CD > 3a) POC early on POD1 with high NPV. On POD3, IL-6 is superior to CRP in terms of high positive predictive power of severe POC. To our knowledge, no study has investigated the advantage of IL-6 on POD1 as early predictor of the need for antibiotic treatment, ICU stay and hospital stay, which is comparable to the CRP serum level late on the third POD. It allows early prediction and could help decide which patients will not potentially benefit from prolonged ATB treatment, and it can guide ICU and hospital discharge. Surgeons should be aware of the need to initiate more intensive care when detecting high IL-6 values, as it could improve the severe course of early postoperative recovery.

### Supplementary Information

**Additional file 1:** **Supplementary document 1:** Figure 1a ROC analysis of predictors of Dindo-Clavien > 3a. Figure 1b ROC analysis of predictors of Dindo-Clavien > 2. Figure 1c ROC analysis of predictors of ICU > 5 days. Figure 1d ROC analysis of predictors of hospital stay > 10 days. Figure 1e ROC analysis of predictors of ATB treatment. Figure 1f ROC analysis of predictors of inflammatory complication. Figure 2a Comparison of predictors values between patients according to Dindo-Clavien > 3a. Figure 2b Comparison of predictors values between patients according to Dindo-Clavien > 2. Figure 2c Comparison of predictors values between patients according to ICU > 5 days. Figure 2d Comparison of predictors values between patients according to hospital stay > 10 days. Figure 2e Comparison of predictors values between patients according to ATB treatment. Figure 2f Comparison of predictors values between patients according to inflammatory complication.

**Additional file 2:** **Supplementary document 2**: Table 1. Comparation of characteristics of patients according to presence of endpoints and their prediction power. Table 2. Comparison of binarized characteristics of patients (cut-off derived employing ROC analysis) according to presence of endpoints and their prediction power. Table 3 IL-6 levels depending on height of rectal tumour.

**Additional file 3:** **Supplementary document 3**: Fig. 1. Section of adenocarcinoma of the rectum from a male patient (T3 N2 M0, grade 3). Negative control shows the specificity of the reaction (A). Positivity for αSMA (B), IL-6 (C) and IL-6R (D) is also demonstrated. IL-6-positive leukocytes are present in the vessel (E). The tumour is infiltrated by IL-6-positive leukocytes (F) that are also on the surface of the tumour tissue (G). These cells also exhibited IL-6R (H). The bar is 300 µm.

## Data Availability

All the source data are stored and can be accessed upon request by corresponding authors.

## Abbreviations

POC: Postoperative complications
IL-6: Interleukin 6
POD: Postoperative day
CRP: C-reactive protein
CD: Clavien-Dindo
ATB: Antibiotics
ICU: Intensive care unit
ROC: Receiver-operating characteristic
AUC: Area under the curve
ERAS: Enhanced recovery after surgery
AL: Anastomotic leakage
CARS: Compensatory anti-inflammatory response syndrome
IBD: Inflammatory bowel disease
MSI: Microsatellite instability
PET-MRI: Positron emission tomography with magnetic resonance imaging
CT: Computed tomography
TME: Total mesorectal excision
PME: Partial mesorectal excision
CRT: Chemoradiotherapy
ICG: Indocyanine green
PIC: Postoperative inflammatory complications
SSI: Surgical site infections
AUROC: Area under the receiver operating characteristic curve
NPV: Negative predictive value
PPV: Positive predictive value
PCT: Procalcitonin
CXCL6: C–X–C motif chemokine ligand 6
CCL11: C–C motif chemokine ligand 11
SN: Sensitivity
SP: Specificity
OR: Odds ratio
