# Supplementary figures and images for: Serum concentrations of proinflammatory biomarker interleukin-6 (IL-6) as a predictor of postoperative complications after elective colorectal surgery
Source: World J Surg Oncol. 2023 Dec 14;21:384. doi: 10.1186/s12957-023-03270-9 (PMC10720211; doi:10.1186/s12957-023-03270-9)

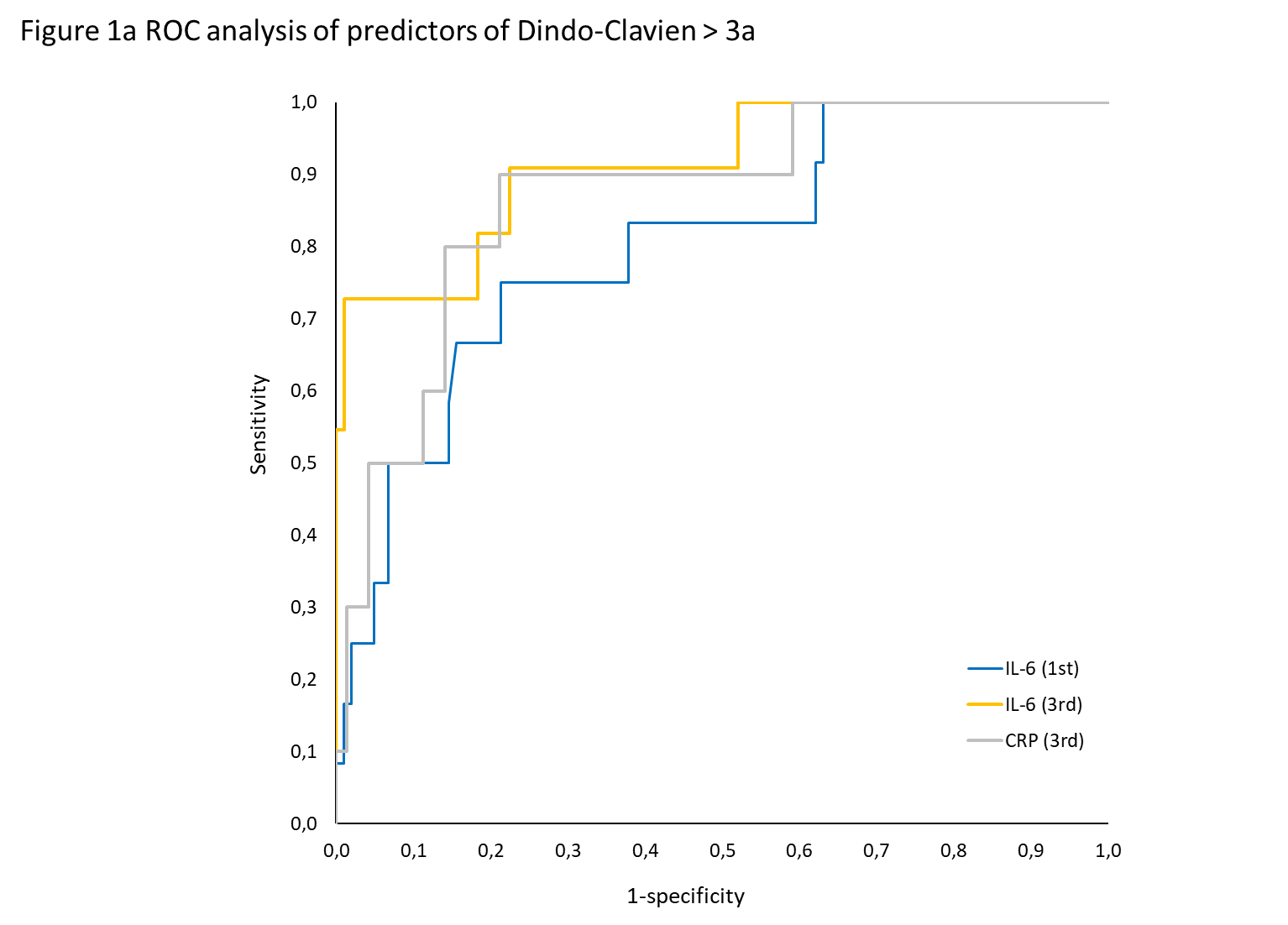

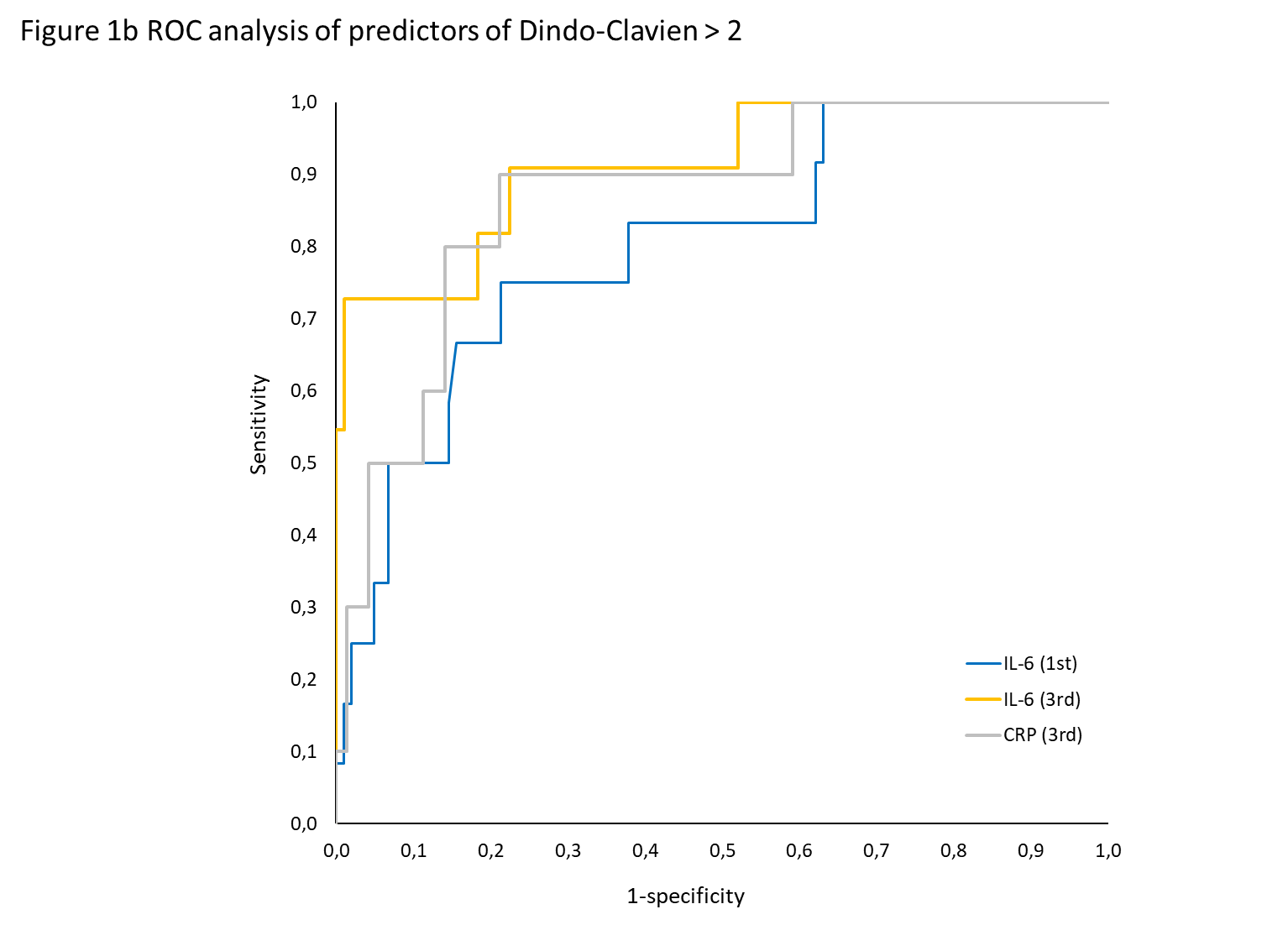


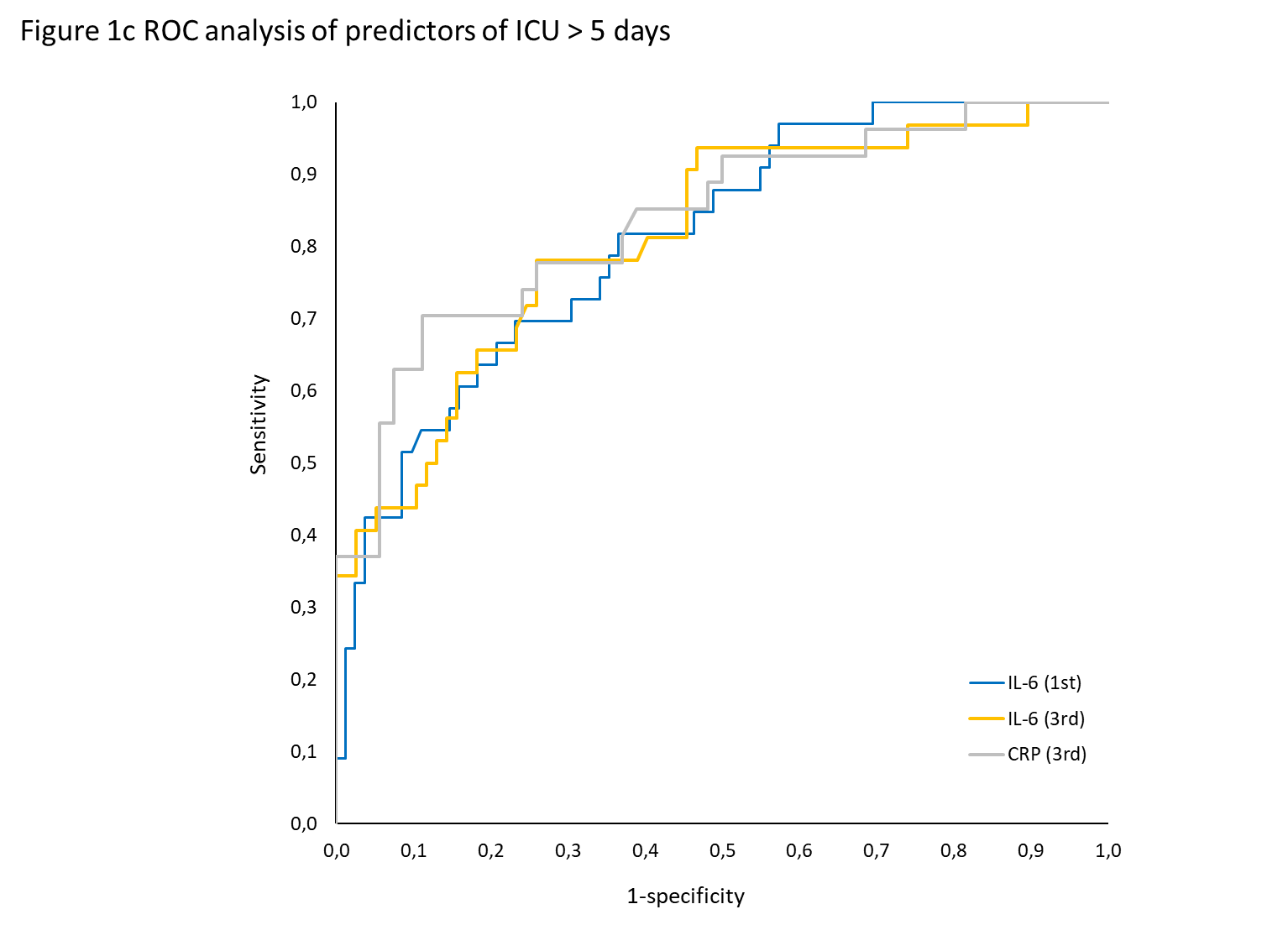


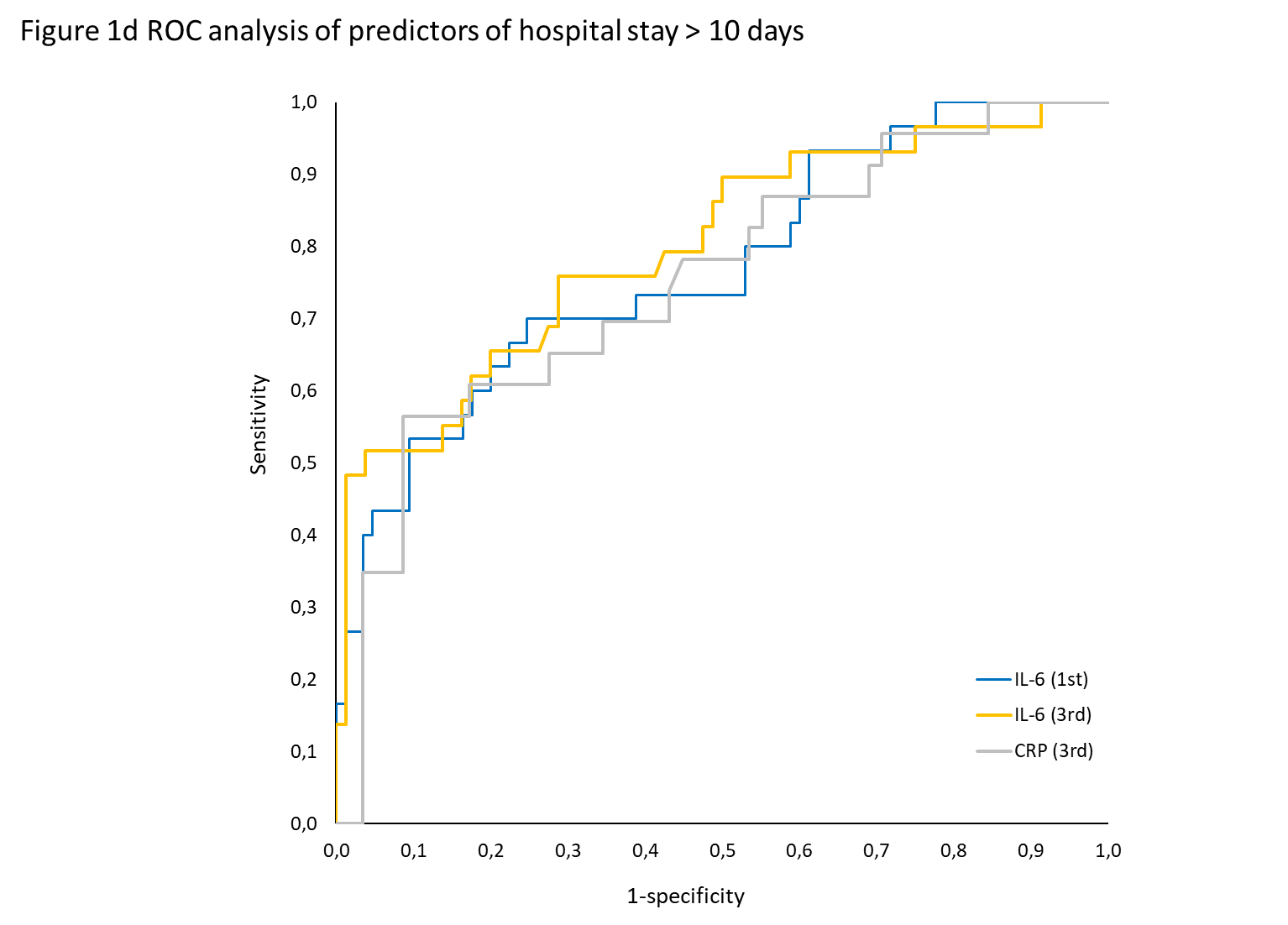


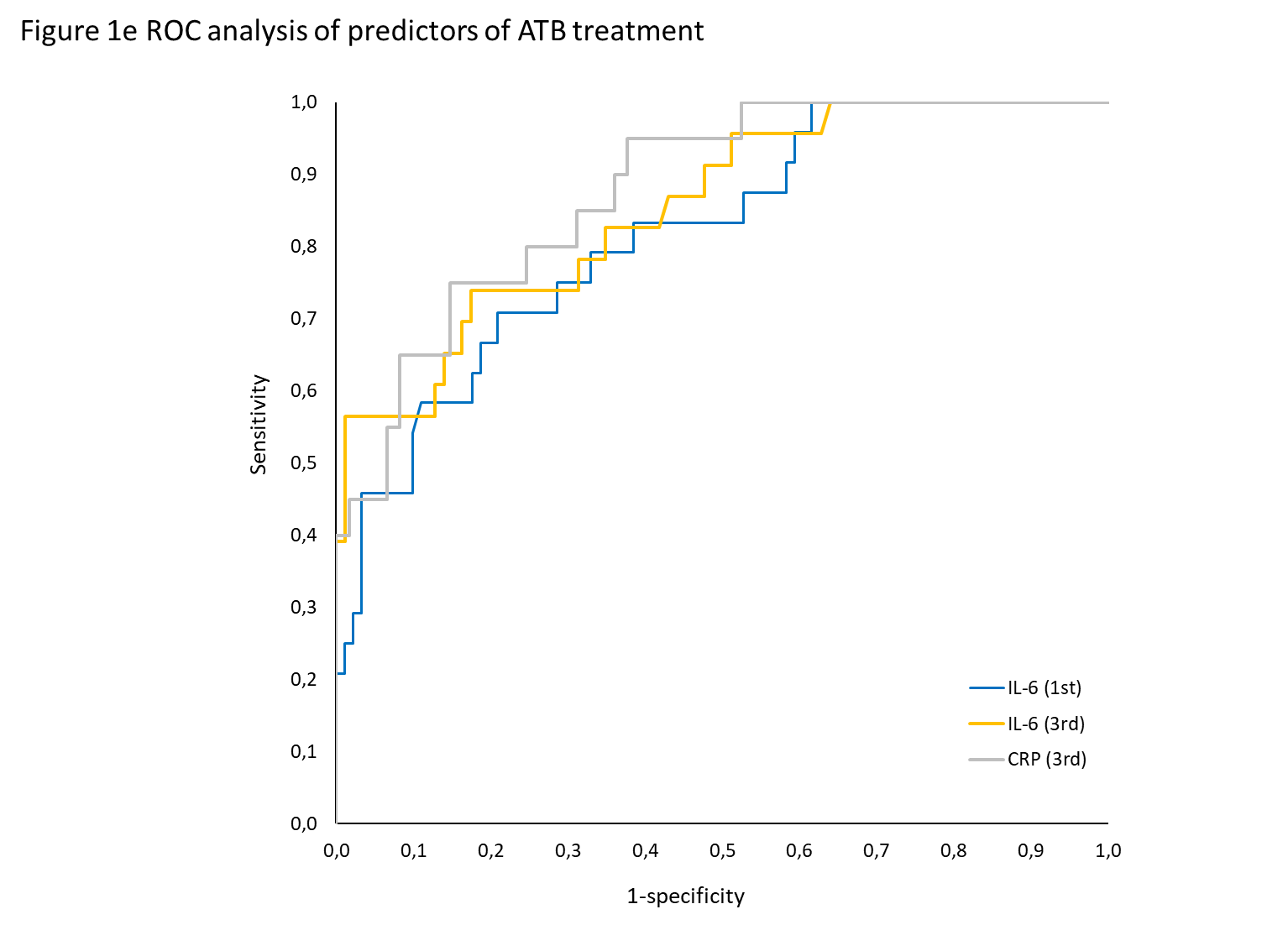

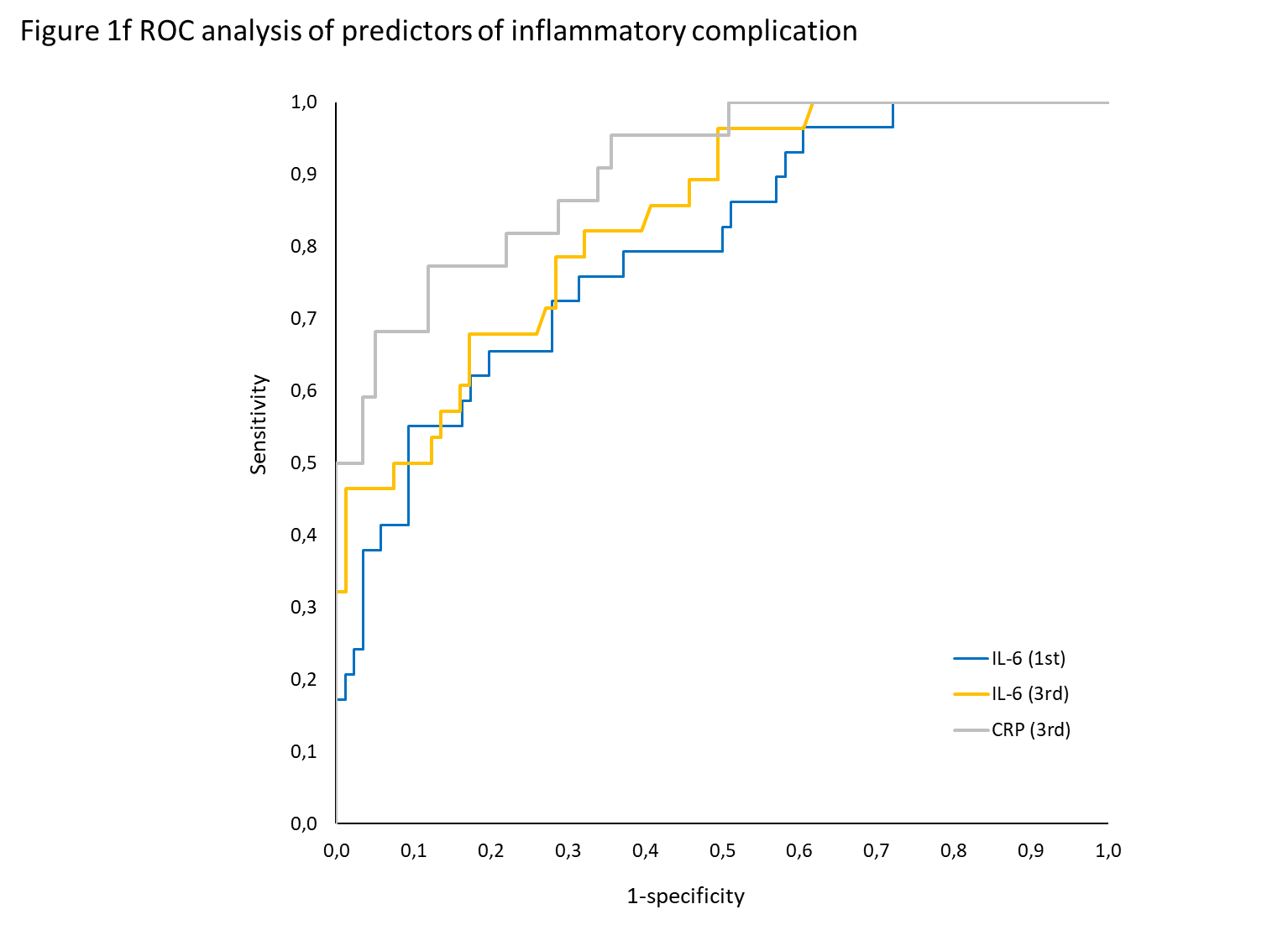


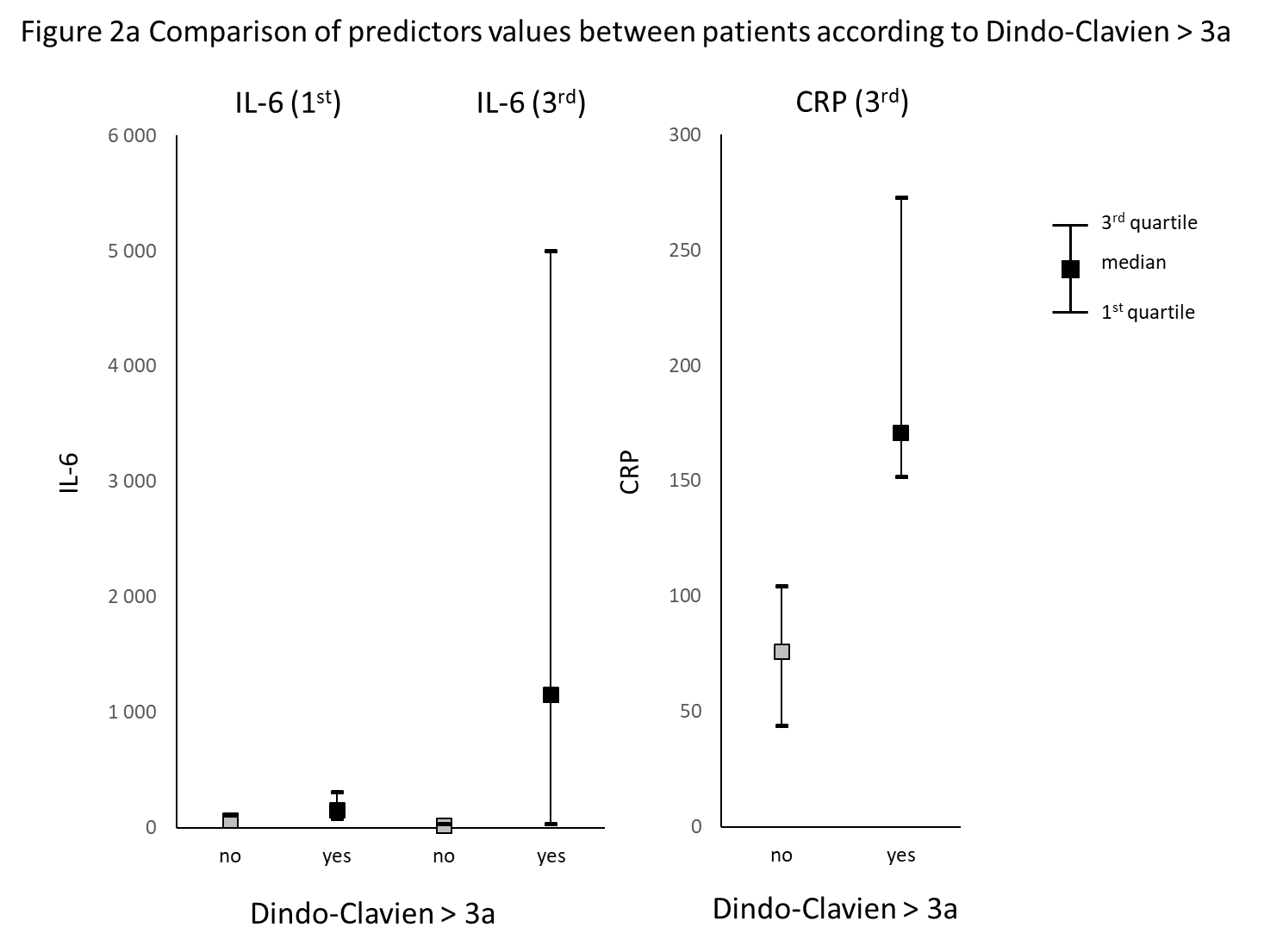


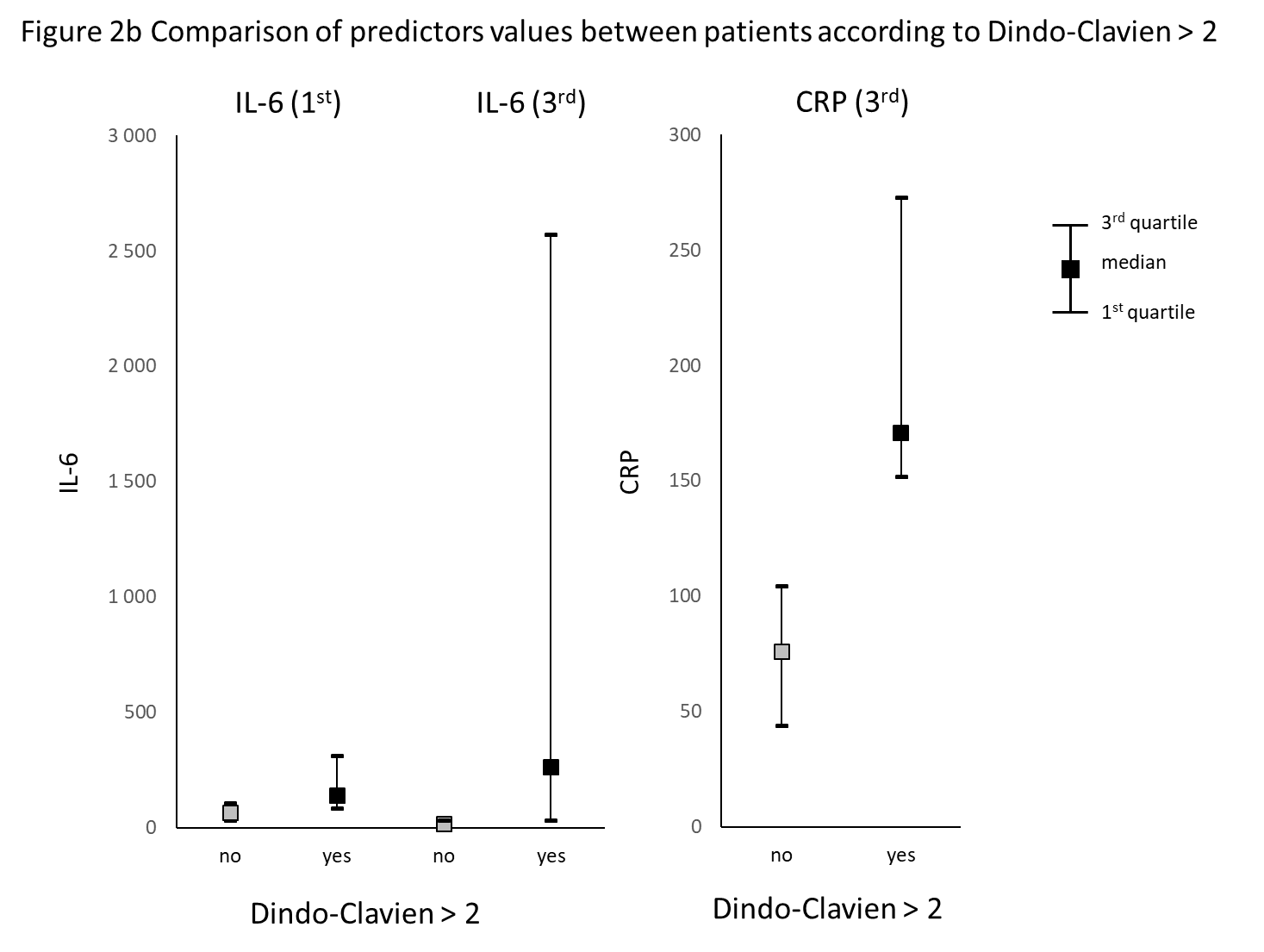


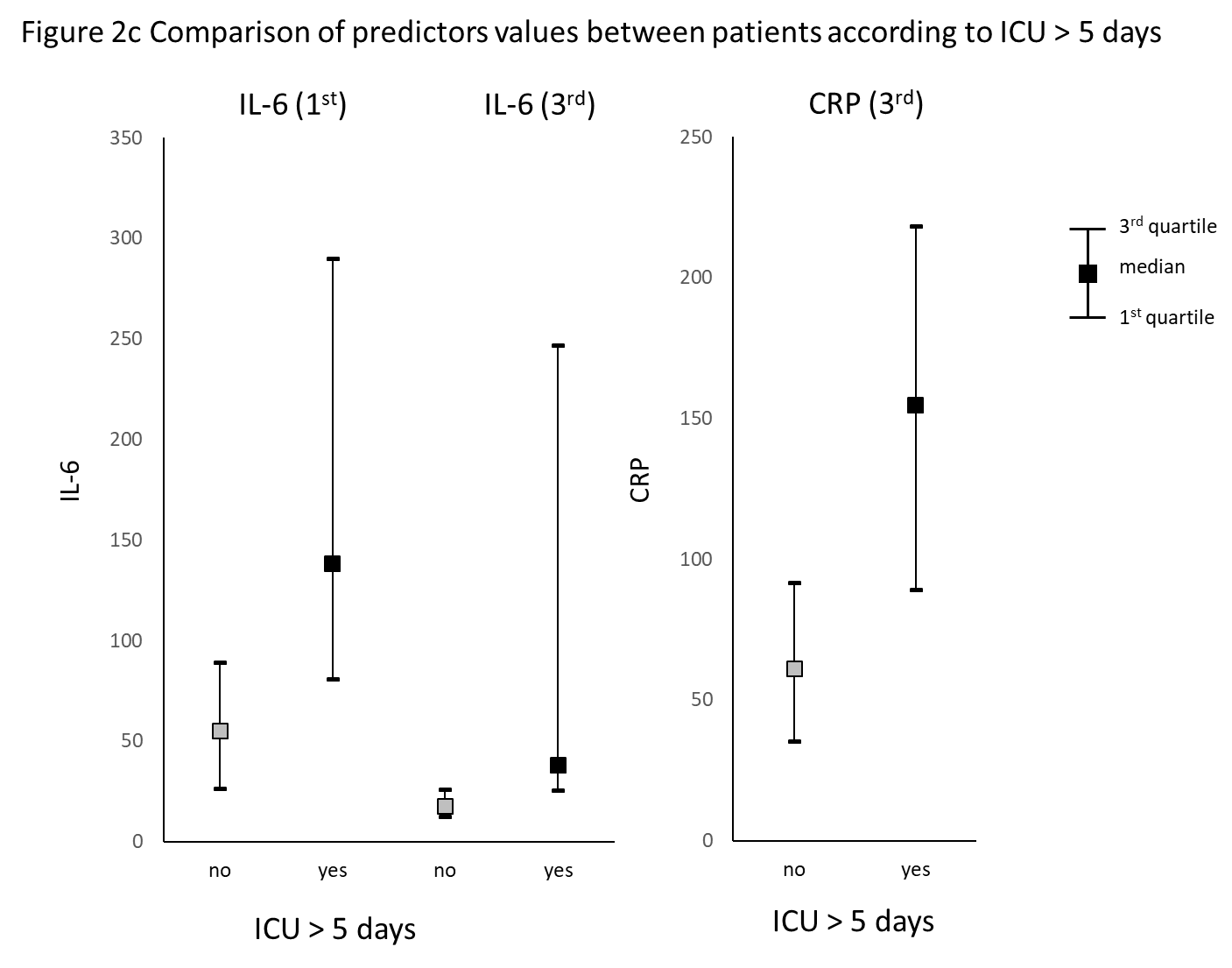


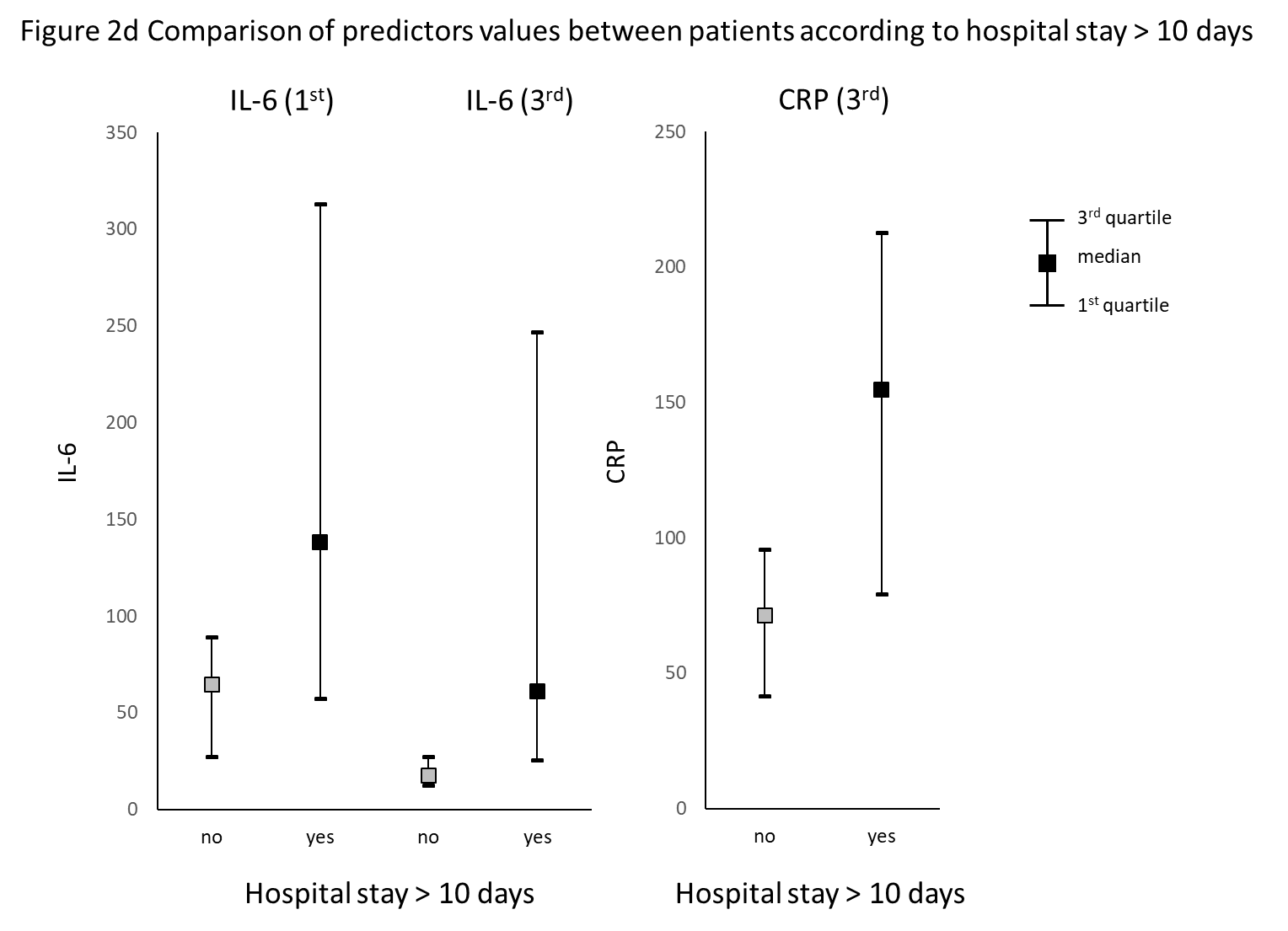


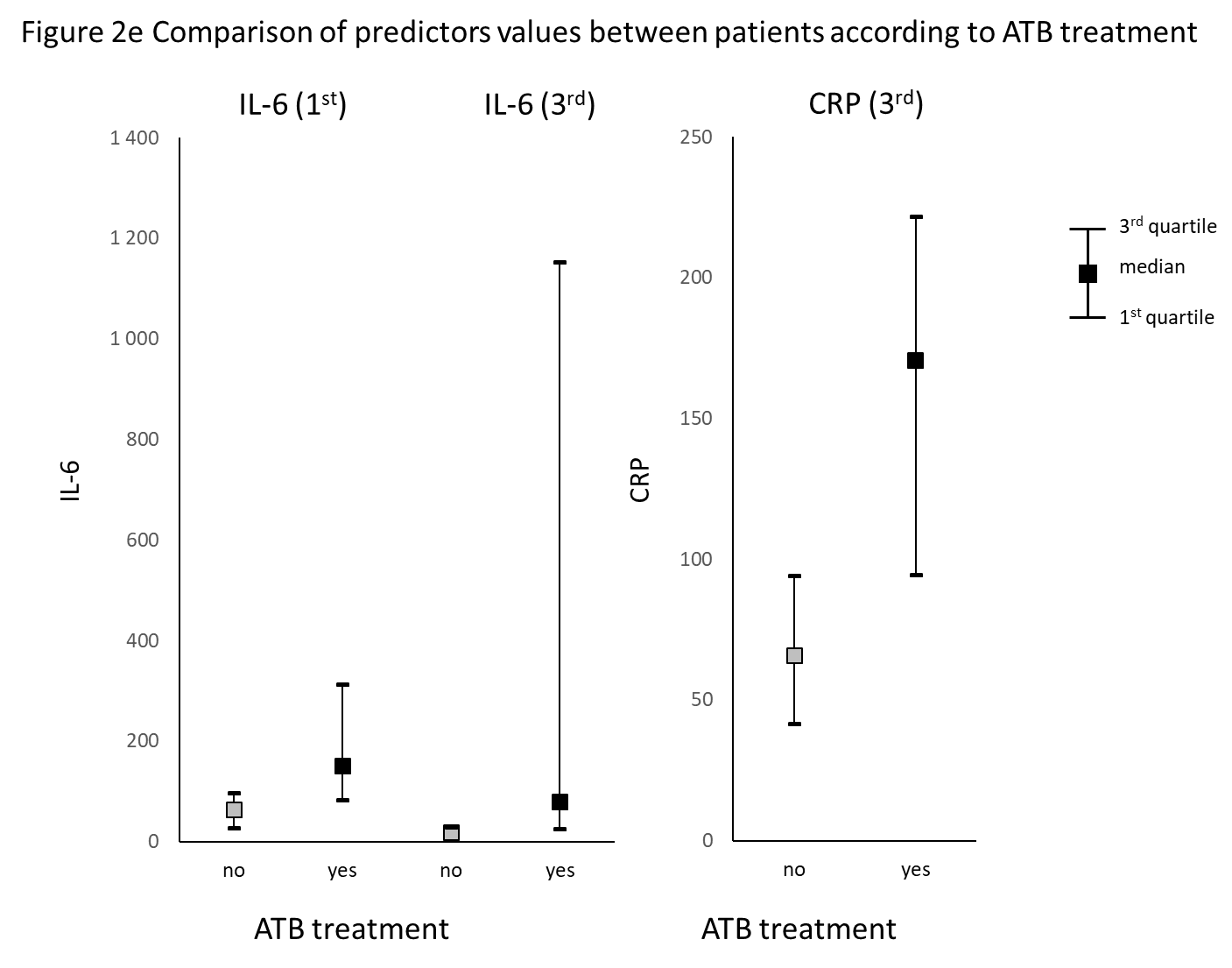


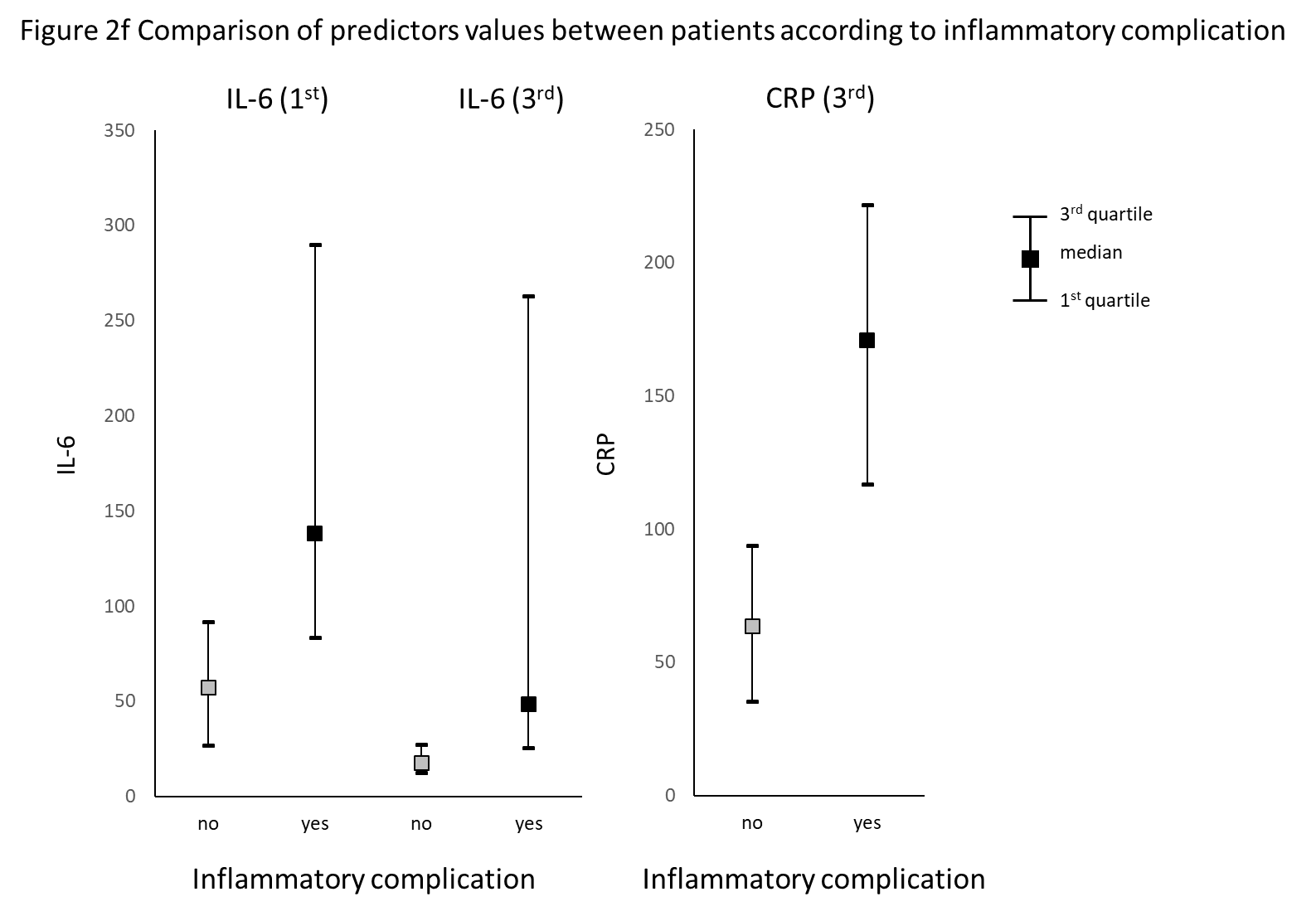

Supplement: Supplementary file 1 — Additional file 1: Supplementary document 1: Figure 1a ROC analysis of predictors of Dindo-Clavien > 3a. Figure 1b ROC analysis of predictors of Dindo-Clavien > 2. Figure 1c ROC analysis of predictors of ICU > 5 days. Figure 1d ROC analysis of predictors of hospital stay > 10 days. Figure 1e ROC analysis of predictors of ATB treatment. Figure 1f ROC analysis of predictors of inflammatory complication. Figure 2a Comparison of predictors values between patients according to Dindo-Clavien > 3a. Figure 2b Comparison of predictors values between patients according to Dindo-Clavien > 2. Figure 2c Comparison of predictors values between patients according to ICU > 5 days. Figure 2d Comparison of predictors values between patients according to hospital stay > 10 days. Figure 2e Comparison of predictors values between patients according to ATB treatment. Figure 2f Comparison of predictors values between patients according to inflammatory complication. [file 12957_2023_3270_MOESM1_ESM.doc]
