# Supplementary material for: Serum concentrations of proinflammatory biomarker interleukin-6 (IL-6) as a predictor of postoperative complications after elective colorectal surgery
Source: World J Surg Oncol. 2023 Dec 14;21:384. doi: 10.1186/s12957-023-03270-9 (PMC10720211; doi:10.1186/s12957-023-03270-9)
Supplement: Supplementary file 2 — Additional file 2: Supplementary document 2: Table 1. Comparation of characteristics of patients according to presence of endpoints and their prediction power. Table 2. Comparison of binarized characteristics of patients (cut-off derived employing ROC analysis) according to presence of endpoints and their prediction power. Table 3 IL-6 levels depending on height of rectal tumour. [file 12957_2023_3270_MOESM2_ESM.doc]

| **Table 1** Comparation of characteristics of patients according to presence of endpoints and their prediction power | | | | |
| --- | --- | --- | --- | --- |
|  | | | | |
|  | **Dindo-Clavien > 3a** | |  |  |
| **Predictor** | **no** | **yes** | **AUC (95% CI)** | **p** |
| IL6 before the surgery | N = 21; 4 (1; 148) | N = 4; 4 (2; 10) | 0.577 (0.272; 0.883) | 0.630 |
| IL6 1^st^ postoperative day | N = 103; 66 (9; 936) | N = 12; 189 (46; 2 746) | 0.804 (0.673; 0.934) | <0.001 |
| IL6 3^rd^ postoperative day | N = 98; 19 (3; 473) | N = 11; 1 153 (19; 50 000) | 0.914 (0.817; 1.000) | <0.001 |
| CRP 3^rd^ posoperative day | N = 71; 76 (14; 342) | N = 10; 192 (63; 420) | 0.869 (0.755; 0.983) | <0.001 |
|  | | | | |
|  | **Dindo-Clavien > 2** | |  |  |
| **Predictor** | **no** | **yes** | **AUC (95% CI)** | **p** |
| IL6 before the surgery | N = 21; 4 (1; 148) | N = 4; 4 (2; 10) | 0.577 (0.272; 0.883) | 0.630 |
| IL6 1^st^ postoperative day | N = 101; 66 (9; 936) | N = 14; 145 (31; 2 746) | 0.751 (0.612; 0.890) | 0.002 |
| IL6 3^rd^ postoperative day | N = 96; 19 (3; 473) | N = 13; 263 (18; 50 000) | 0.865 (0.755; 0.976) | <0.001 |
| CRP 3^rd^ postoperative day | N = 71; 76 (14; 342) | N = 10; 192 (63; 420) | 0.869 (0.755; 0.983) | <0.001 |
|  | | | | |
|  | **ICU > 5 days** | |  |  |
| **Predictor** | **no** | **yes** | **AUC (95% CI)** | **p** |
| IL6 before the surgery | N = 16; 3 (2; 30) | N = 9; 6 (1; 148) | 0.514 (0.239; 0.789) | 0.910 |
| IL6 1^st^ postoperative day | N = 82; 56 (9; 419) | N = 33; 138 (32; 2 746) | 0.811 (0.727; 0.895) | <0.001 |
| IL6 3^rd^ postoperative day | N = 77; 18 (3; 83) | N = 32; 43 (9; 50 000) | 0.814 (0.724; 0.904) | <0.001 |
| CRP 3^rd^ postoperative day | N = 54; 62 (14; 185) | N = 27; 155 (32; 420) | 0.838 (0.742; 0.934) | <0.001 |
|  | | | | |
|  | **Hospitalization > 10 days** | |  |  |
| **Predictor** | **no** | **yes** | **AUC (95% CI)** | **p** |
| IL6 before the surgery | N = 17; 4 (2; 30) | N = 8; 4 (1; 148) | 0.559 (0.270; 0.848) | 0.641 |
| IL6 1^st^ postoperative day | N = 85; 65 (9; 352) | N = 30; 138 (26; 2 746) | 0.771 (0.669; 0.872) | <0.001 |
| IL6 3^rd^ postoperative day | N = 80; 18 (3; 1 387) | N = 29; 61 (8; 50 000) | 0.800 (0.700; 0.899) | <0.001 |
| CRP 3^rd^ postoperative day | N = 58; 73 (14; 420) | N = 23; 155 (32; 292) | 0.752 (0.631; 0.873) | <0.001 |
|  | | | | |
|  | **ATB therapy** | |  |  |
| **Predictor** | **no** | **yes** | **AUC (95% CI)** | **p** |
| IL6 before the surgery | N = 19; 4 (2; 148) | N = 6; 2 (1; 10) | 0.746 (0.492; 1.000) | 0.075 |
| IL6 1^st^ postoperative day | N = 91; 64 (9; 352) | N = 24; 152 (43; 2 746) | 0.818 (0.725; 0.912) | <0.001 |
| IL6 3^rd^ postoperative day | N = 86; 18 (3; 114) | N = 23; 79 (15; 50 000) | 0.854 (0.766; 0.942) | <0.001 |
| CRP ^3rd^ postoperative day | N = 61; 66 (14; 201) | N = 20; 171 (63; 420) | 0.879 (0.798; 0.959) | <0.001 |
|  |  |  |  |  |
|  | **Inflammatory complications** | |  |  |
| **Predictor** | **no** | **yes** | **AUC (95% CI)** | **p** |
| IL6 before the surgery | N = 18; 4 (2; 148) | N = 7; 2 (1; 10) | 0.659 (0.387; 0.930) | 0.226 |
| IL6 ^1st^ postoperative day | N = 86; 61 (9; 352) | N = 29; 138 (31; 2 746) | 0.798 (0.705; 0.890) | <0.001 |
| IL6 3^rd^ postoperative day | N = 81; 18 (3; 114) | N = 28; 52 (15; 50 000) | 0.839 (0.758; 0.920) | <0.001 |
| CRP 3^rd^ postoperative day | N = 59; 64 (14; 178) | N = 22; 178 (63; 420) | 0.904 (0.833; 0.974) | <0.001 |

| **Table 2** Comparison of binarized characteristics of patients (cut-off derived employing ROC analysis) according to presence of endpoints and their prediction power | | | | | | | | | | |
| --- | --- | --- | --- | --- | --- | --- | --- | --- | --- | --- |
| **Dindo-Clavien > 3a** | | | | | | | | | | |
|  | **no** |  | **yes** |  |  |  |  |  |  |  |
| **Predictor cut-off** | **< cut-off** | **≥ cut off** | **< cut-off** | **≥ cut off** | **AUC (95% CI)** | **p** | **Sensitivity** | **Specificity** | **NPV** | **PPV** |
| **IL6 before the surgery ≥ 5.0** | N=17 (81.0%) | N=4 (19.0%) | N=2 (50.0%) | N=2 (50.0%) | 0.655 (0.335; 0.974) | 0.335 | 50,0% | 81,0% | 89,5% | 33,3% |
| **IL6 1^st^ postoperative day ≥ 113.0** | N=81 (78.6%) | N=22 (21.4%) | N=3 (25.0%) | N=9 (75.0%) | 0.768 (0.619; 0.918) | 0.002 | 75,0% | 78,6% | 96,4% | 29,0% |
| **IL6 3^rd^ postoperative day ≥ 180.5** | N=97 (99.0%) | N=1 (1.0%) | N=3 (27.3%) | N=8 (72.7%) | 0.859 (0.699; 1.000) | <0.001 | 72,7% | 99,0% | 97,0% | 88,9% |
| **CRP 3^rd^ postoperative day ≥ 113.8** | N=56 (78.9%) | N=15 (21.1%) | N=1 (10.0%) | N=9 (90.0%) | 0.844 (0.721; 0.968) | <0.001 | 90,0% | 78,9% | 98,2% | 37,5% |
|  | | | | | | | | | | |
| **Dindo-Clavien > 2** | | | | | | | | | | |
|  | **no** |  | **yes** |  |  |  |  |  |  |  |
| **Predictor cut-off** | **< cut-off** | **≥ cut off** | **< cut-off** | **≥ cut off** | **AUC (95% CI)** | **p** | **Sensitivity** | **Specificity** | **NPV** | **PPV** |
| **IL6 before the surgery ≥ 5.0** | N=17 (81.0%) | N=4 (19.0%) | N=2 (50.0%) | N=2 (50.0%) | 0.655 (0.335; 0.974) | 0.335 | 50,0% | 81,0% | 89,5% | 33,3% |
| **IL6 1^st^ postoperative day ≥ 113.0** | N=79 (78.2%) | N=22 (21.8%) | N=5 (35.7%) | N=9 (64.3%) | 0.713 (0.558; 0.867) | 0.010 | 64,3% | 78,2% | 94,0% | 29,0% |
| **IL6 3^rd^ postoperative day ≥ 34.0** | N=74 (77.1%) | N=22 (22.9%) | N=2 (15.4%) | N=11 (84.6%) | 0.808 (0.684; 0.933) | <0.001 | 84,6% | 77,1% | 97,4% | 33,3% |
| **CRP 3^rd^ postoperative day ≥ 113.8** | N=56 (78.9%) | N=15 (21.1%) | N=1 (10.0%) | N=9 (90.0%) | 0.844 (0.721; 0.968) | <0.001 | 90,0% | 78,9% | 98,2% | 37,5% |
|  | | | | | | | | | | |
| **ICU > 5 dní** | | | | | | | | | | |
|  | **no** |  | **yes** |  |  |  |  |  |  |  |
| **Predictor cut-off** | **< cut-off** | **≥ cut off** | **< cut-off** | **≥ cut off** | **AUC (95% CI)** | **p** | **Sensitivity** | **Specificity** | **NPV** | **PPV** |
| **IL6 before the surgery ≥ 7.5** | N=14 (87.5%) | N=2 (12.5%) | N=5 (55.6%) | N=4 (44.4%) | 0.660 (0.423; 0.897) | 0.193 | 44,4% | 87,5% | 73,7% | 66,7% |
| **IL6 1^st^ postoperative day ≥ 89.3** | N=63 (76.8%) | N=19 (23.2%) | N=10 (30.3%) | N=23 (69.7%) | 0.733 (0.627; 0.838) | <0.001 | 69,7% | 76,8% | 86,3% | 54,8% |
| **IL6 3. den po operaci ≥ 25.0** | N=57 (74.0%) | N=20 (26.0%) | N=7 (21.9%) | N=25 (78.1%) | 0.761 (0.660; 0.862) | <0.001 | 78,1% | 74,0% | 89,1% | 55,6% |
| **CRP 3. den po operaci ≥ 109.0** | N=48 (88.9%) | N=6 (11.1%) | N=8 (29.6%) | N=19 (70.4%) | 0.796 (0.682; 0.910) | <0.001 | 70,4% | 88,9% | 85,7% | 76,0% |
|  | | | | | | | | | | |
| **Hospitalisation > 10 dní** | | | | | | | | | | |
|  | **no** |  | **yes** |  |  |  |  |  |  |  |
| **Predictor cut-off** | **< cut-off** | **≥ cut off** | **< cut-off** | **≥ cut off** | **AUC (95% CI)** | **p** | **Sensitivity** | **Specificity** | **NPV** | **PPV** |
| **IL6 before the surgery ≥ 8.8** | N=15 (88.2%) | N=2 (11.8%) | N=4 (50.0%) | N=4 (50.0%) | 0.691 (0.449; 0.933) | 0.130 | 50,0% | 88,2% | 78,9% | 66,7% |
| **IL6 1^st^ postoperative day ≥ 89.3** | N=64 (75.3%) | N=21 (24.7%) | N=9 (30.0%) | N=21 (70.0%) | 0.726 (0.617; 0.836) | <0.001 | 70,0% | 75,3% | 87,7% | 50,0% |
| **IL6 3^rd^ postoperative day ≥ 60.9** | N=77 (96.3%) | N=3 (3.8%) | N=14 (48.3%) | N=15 (51.7%) | 0.740 (0.619; 0.861) | <0.001 | 51,7% | 96,3% | 84,6% | 83,3% |
| **CRP 3^rd^ postoperative day ≥ 146.0** | N=53 (91.4%) | N=5 (8.6%) | N=10 (43.5%) | N=13 (56.5%) | 0.740 (0.605; 0.874) | <0.001 | 56,5% | 91,4% | 84,1% | 72,2% |
|  | | | | | | | | | | |
| **ATB therapy** | | | | | | | | | | |
|  | **no** |  | **yes** |  |  |  |  |  |  |  |
| **Predictor cut-off** | **< cut-off** | **≥ cut off** | **< cut-off** | **≥ cut off** | **AUC (95% CI)** | **p** | **Sensitivity** | **Specificity** | **NPV** | **PPV** |
| **IL6 before the surgery ≥ 0.0** | N=17 (89.5%) | N=2 (10.5%) | N=2 (33.3%) | N=4 (66.7%) | 0.781 (0.537; 1.000) | 0.042 | 66,7% | 89,5% | 89,5% | 66,7% |
| **IL6 1^st^ postoperative day ≥ 104.0** | N=72 (79.1%) | N=19 (20.9%) | N=7 (29.2%) | N=17 (70.8%) | 0.750 (0.633; 0.866) | <0.001 | 70,8% | 79,1% | 91,1% | 47,2% |
| **IL6 3^rd^ postoperative day≥ 34.2** | N=71 (82.6%) | N=15 (17.4%) | N=6 (26.1%) | N=17 (73.9%) | 0.782 (0.668; 0.897) | <0.001 | 73,9% | 82,6% | 92,2% | 53,1% |
| **CRP 3^rd^ postoperative day ≥ 113.8** | N=52 (85.2%) | N=9 (14.8%) | N=5 (25.0%) | N=15 (75.0%) | 0.801 (0.679; 0.924) | <0.001 | 75,0% | 85,2% | 91,2% | 62,5% |
|  | | | | | | | | | | |
| **Inflammatory complications** | | | | | | | | | | |
|  | **no** |  | **yes** |  |  |  |  |  |  |  |
| **Predictor cut-off** | **< cut-off** | **≥ cut off** | **< cut-off** | **≥ cut off** | **AUC (95% CI)** | **p** | **Sensitivity** | **Specificity** | **NPV** | **PPV** |
| **IL6 before the surgery ≥ 8.8** | N=16 (88.9%) | N=2 (11.1%) | N=3 (42.9%) | N=4 (57.1%) | 0.730 (0.485; 0.976) | 0.079 | 57,1% | 88,9% | 84,2% | 66,7% |
| **IL6 1^st^ postoperative day ≥ 136.5** | N=78 (90.7%) | N=8 (9.3%) | N=13 (44.8%) | N=16 (55.2%) | 0.729 (0.610; 0.848) | <0.001 | 55,2% | 90,7% | 85,7% | 66,7% |
| **IL6 3^rd^ postoperative day ≥ 34.0** | N=67 (82.7%) | N=14 (17.3%) | N=9 (32.1%) | N=19 (67.9%) | 0.753 (0.640; 0.865) | <0.001 | 67,9% | 82,7% | 88,2% | 57,6% |
| **CRP 3^rd^ postoperative day ≥ 113.8** | N=52 (88.1%) | N=7 (11.9%) | N=5 (22.7%) | N=17 (77.3%) | 0.827 (0.714; 0.940) | <0.001 | 77,3% | 88,1% | 91,2% | 70,8% |

| **Table 3** IL-6 levels depending on height of rectal tumor | | | |
| --- | --- | --- | --- |
| upper rectum | | | |
|  | no | yes | p |
| **IL6 1^st^ postoperative day** | N=20; 52.8 (12.7; 419.0) | N=24; 65.8 (9.2; 936.0) | 0.346 |
| **IL6 3^rd^ postoperative day** | N=20; 17.9 (5.8; 109.0) | N=22; 19.8 (3.0; 76.8) | 0.489 |
| middle | | | |
|  | no | yes | p |
| **IL6 1^st^ postoperative day** | N=34; 62.8 (9.2; 936.0) | N=10; 43.5 (13.9; 346.0) | 0.431 |
| **IL6 3^rd^ postoperative day** | N=32; 19.1 (3.0; 78.9) | N=10; 18.8 (11.7; 109.0) | 0.652 |
| lower | | | |
|  | no | yes | p |
| **IL6 1^st^ postoperative day** | N=34; 51.1 (9.2; 936.0) | N=10; 62.8 (12.7; 419.0) | 0.772 |
| **IL6 3^rd^ postoperative day** | N=32; 19.8 (3.0; 109.0) | N=10; 17.4 (5.8; 78.9) | 0.213 |
