# Supplementary material for: Serum concentrations of proinflammatory biomarker interleukin-6 (IL-6) as a predictor of postoperative complications after elective colorectal surgery
Source: World J Surg Oncol. 2023 Dec 14;21:384. doi: 10.1186/s12957-023-03270-9 (PMC10720211; doi:10.1186/s12957-023-03270-9)

Detection of IL-6 in resected tissue - method

For immunohistochemical analysis, formaldehyde-fixed paraffin-embedded tumour samples from randomly selected patients were sectioned (5μm thickness). After conventional dewaxing (xylene-to-alcohol, 5 minutes for each bath), the sections were rehydrated with distilled water. Heat-induced epitope retrieval was performed in citrate buffer (at pH 6.0) using a pressure cooker.^22^ To quench endogenous peroxidase activity, the sections were incubated in 1% hydrogen peroxide solution (in PBS) for 20 minutes at room temperature. To avoid non-specific reactivity, sections were treated in 5% Roti-ImmunoBlock (CarlRoth, Karlsruhe, Germany). Primary antibodies (rabbit polyclonal anti-IL-6R antibody (ab128008), mouse monoclonal anti-IL-6  antibody, [clone 1.2-2B11-2G10], (ab 9324) -  both Abcam, Cambridge, UK; mouse monoclonal anti-SMA [clone 1A4], Dako, Glostrup, Denmark)  were diluted 1/100 in DAKO-Real antibody diluent and incubated overnight at 4°C. Negative controls were performed by omitting the primary antibody, and appropriate isotype controls were applied instead (rabbit ab172730; mouse ab37355 - both Abcam) in identical dilution.

After profound washing in running water, the secondary polymer HRP-tagged antibody (Histofine® Simple Stain™ M.A.X. PO MULTI; Nichirei Biosciences, Tokyo, Japan) was incubated for 30 minutes. Chromogenic detection was performed using Histofine® Simple Stain™ A.E.C. Solution. Slides were counterstained in Gill's haematoxylin and mounted in Biomount Aqua (Baria, Praha, Czech Republic).

## Detection of IL-6 and IL-6R in cancer samples - results

To verify the tumour as the source of IL-6, colorectal carcinoma from patients was subjected to histological analysis. Cells positive for IL-6 and IL-6R were observed in the stroma (adenocarcinoma of the rectum as a typical representative of colorectal cancer is presented in Fig. 1A-H) contrasting to negative control (Fig. 1A). Smooth muscle cells of the muscularis mucosae layer were positive for αSMA (Fig. 1B). Cells positive for IL-6, phenotypically consistent with cancer-associated fibroblasts, migrated from this layer (Fig. 1C). No cells in this section exhibited the presence of IL-6R (Fig. 1D). Another part of the same sample demonstrated a vessel with accumulated leukocytes positive for IL-6 (Fig. 1E). Leukocytic infiltration of the tumour was intensive (Fig. 1F) and on the surface of the tumour (Fig. 1G) expressed both IL-6 and IL-6R (Fig. 1H). Il-6R-positive cells were also detected in the dilated vessels (Fig. 1H).

Fig. 1. Section of adenocarcinoma of the rectum from a male patient (T3 N2 M0, grade 3). Negative control shows the specificity of the reaction (A). Positivity for αSMA (B), IL-6 (C) and IL-6R (D) is also demonstrated. IL-6-positive leukocytes are present in the vessel (E). The tumour is infiltrated by IL-6-positive leukocytes (F) that are also on the surface of the tumour tissue (G). These cells also exhibited IL-6R (H). The bar is 300 µm.


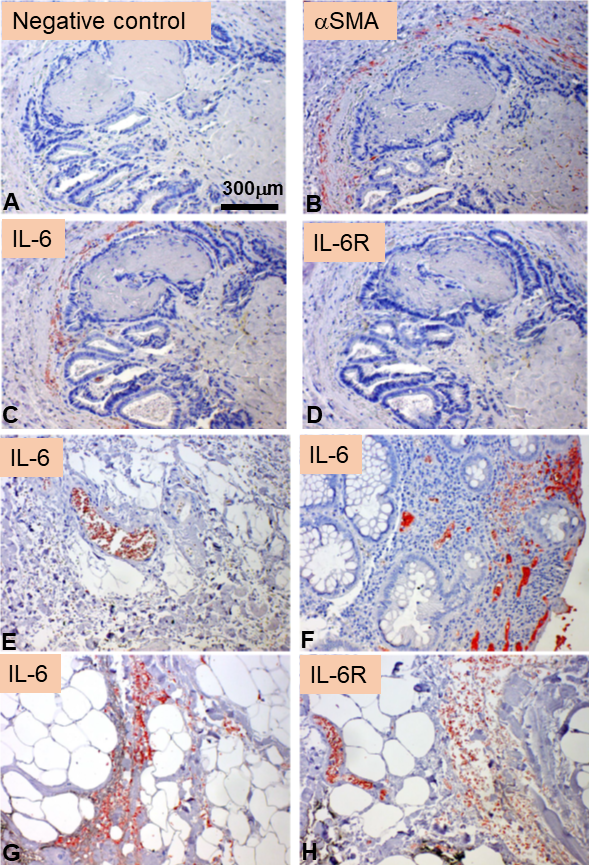

Supplement: Supplementary file 3 — Additional file 3: Supplementary document 3: Fig. 1. Section of adenocarcinoma of the rectum from a male patient (T3 N2 M0, grade 3). Negative control shows the specificity of the reaction (A). Positivity for αSMA (B), IL-6 (C) and IL-6R (D) is also demonstrated. IL-6-positive leukocytes are present in the vessel (E). The tumour is infiltrated by IL-6-positive leukocytes (F) that are also on the surface of the tumour tissue (G). These cells also exhibited IL-6R (H). The bar is 300 µm. [file 12957_2023_3270_MOESM3_ESM.doc]
